# hnRNPs: roles in neurodevelopment and implication for brain disorders

**DOI:** 10.3389/fnmol.2024.1411639

**Published:** 2024-07-17

**Authors:** Pierre Tilliole, Simon Fix, Juliette D. Godin

**Affiliations:** ^1^Institut de Génétique et de Biologie Moléculaire et Cellulaire, IGBMC, Illkirch, France; ^2^Centre National de la Recherche Scientifique, CNRS, UMR7104, Illkirch, France; ^3^Institut National de la Santé et de la Recherche Médicale, INSERM, U1258, Illkirch, France; ^4^Université de Strasbourg, Strasbourg, France

**Keywords:** hnRNP proteins, alternative splicing, brain development, neurodevelopmental disorders, neurodegenerative disorders

## Abstract

Heterogeneous nuclear ribonucleoproteins (hnRNPs) constitute a family of multifunctional RNA-binding proteins able to process nuclear pre-mRNAs into mature mRNAs and regulate gene expression in multiple ways. They comprise at least 20 different members in mammals, named from A (*HNRNP A1*) to U (*HNRNP U*). Many of these proteins are components of the spliceosome complex and can modulate alternative splicing in a tissue-specific manner. Notably, while genes encoding hnRNPs exhibit ubiquitous expression, increasing evidence associate these proteins to various neurodevelopmental and neurodegenerative disorders, such as intellectual disability, epilepsy, microcephaly, amyotrophic lateral sclerosis, or dementias, highlighting their crucial role in the central nervous system. This review explores the evolution of the hnRNPs family, highlighting the emergence of numerous new members within this family, and sheds light on their implications for brain development.

## 1 Introduction

The exact number of protein-coding genes within the human genome remains a subject of intensive discussion, with estimated number that dropped from 30,000 to 40,000 since the initial publication of the human genome (Lander et al., 2001; Venter et al., 2001) to less than 20,000 today (Morales et al., 2022; Nurk et al., 2022). If each gene encoded a single protein, the estimated size of the proteome would be approximately 20,000. However, around 95% of the human multi-exon genes are able to produce multiple protein sequences (Pan et al., 2008; Wang et al., 2008), resulting in a number of distinct human proteins exceeding 70,000 (Aebersold et al., 2018). This extended protein diversity is the result of alternative splicing, a process that generates, in a tissue specific manner, several mRNA transcripts from the same gene. Notably, the brain is one of the organs with the highest number of splicing events (Mazin et al., 2021), making it particularly sensitive to defects in this process (Grabowski and Black, 2001).

The mRNA splicing is a multi-step process catalyzed by various small nuclear ribonucleoprotein (snRNP) particles that dynamically assemble, along with other proteins, in a macromolecular machinery called the spliceosome. Splicing starts with the recognition of specific sequences at the exon-intron boundaries by the spliceosome. Alternative splicing additionally involves cis-acting regulatory sequences, that, through their interaction with trans-acting splicing factors, modulate the activity of nearby splice sites. Splice site selection is followed by two successive transesterification reactions that lead to the removal of the intron and the joining of neighboring exons, ultimately yielding to mature mRNA (reviewed by Wilkinson et al., 2020).

**Figure 1:**
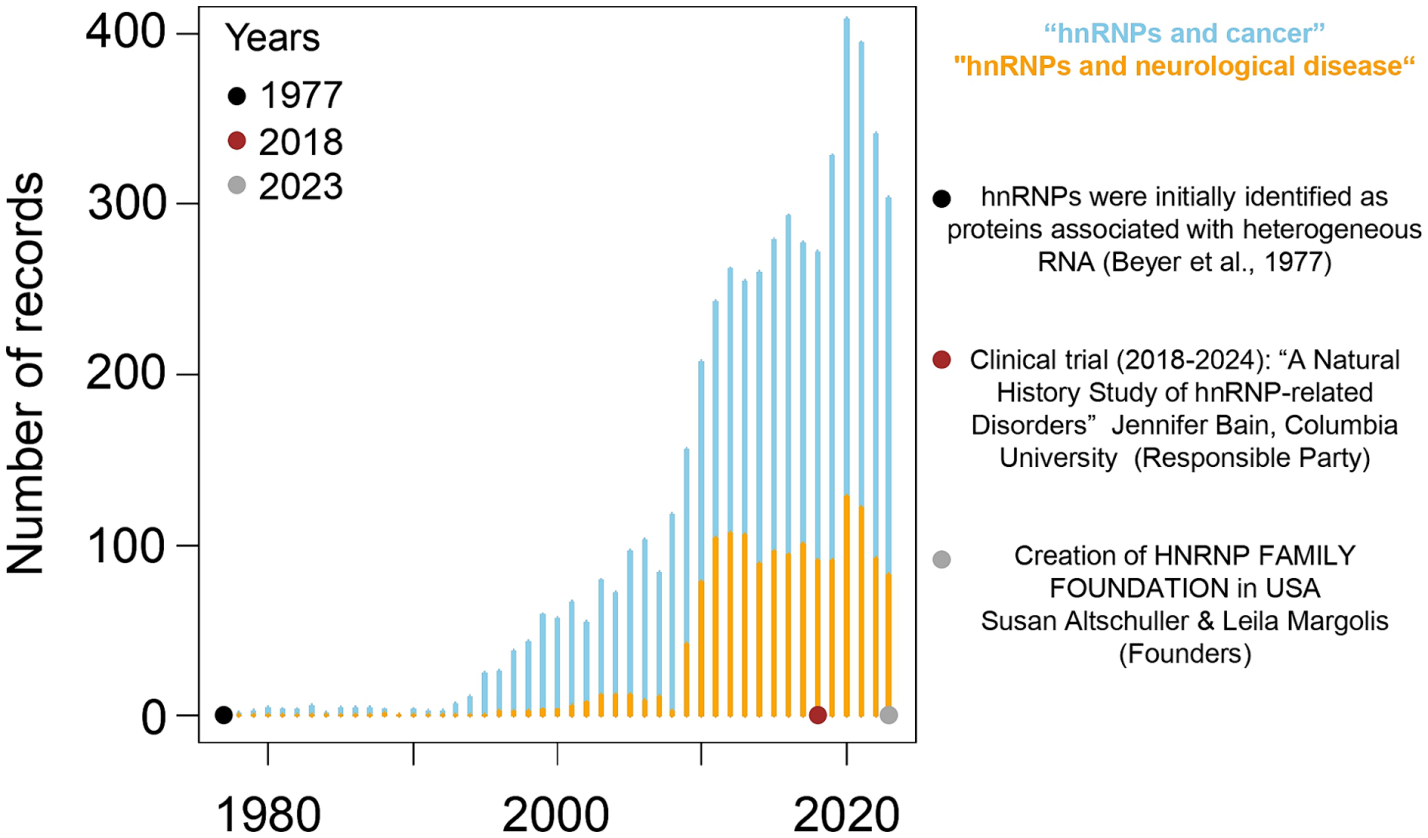
Number of records retrieved from PubMed including keywords “hnRNPs and cancer” or “hnRNPs and neurological disease” in the period 1977–2023. Date of search: March 11, 2024.

Among the two major classes of splicing factors, one finds the heterogeneous nuclear ribonucleoproteins (hnRNPs). hnRNPs constitute a family of 20 canonical multifunctional RNA-binding proteins (RBPs) in mammals, named from A (HNRNP A1) to U (HNRNP U) (Chaudhury et al., 2010). As components of the spliceosomal assembly, these proteins modulate alternative splicing. Strikingly, although genes encoding those canonical hnRNPs are ubiquitously expressed, genetic variants altering their sequence mainly lead to neurodevelopmental or neurodegenerative disorders, such as intellectual disability, epilepsy, microcephaly, amyotrophic lateral sclerosis, or dementias, pointing out their key role in the central nervous system (Purice and Taylor, 2018; Low et al., 2021). Yet, compared to their dysfunction in cancer, the significance of hnRNPs in neurological disorders remains largely unexplored (Figure 1). Nevertheless, two recent developments mark a growing interest in hnRNPs brain-related disorders (Figure 1): (i) the initiation, in 2018, of a clinical study (Natural History Study of hnRNP-related Disorders; ClinicalTrials.gov ID: NCT03492060), that aims to examine neurological traits in individuals with variants in any hnRNP genes with the ultimate goal to define a hnRNP neurodevelopmental syndrome and propose common therapeutic interventions; and (ii) the creation, in 2023 and 2024, of two foundations, the HNRNP Family Foundation in USA^1^ and the HNRNP Japan,^2^ dedicated to support patients and families living with hnRNP-related neurodevelopmental disorders.

In this review, we provide updated insights into the implications of hnRNPs in neurodevelopmental and neurodegenerative disorders, by exploring the evolution of hnRNPs in mammalian genomes, their differential expression and localization and their physiological roles with a particular focus on the developing and aging brain.

## 2 Evolution of the hnRNP family

### 2.1 Identification of the major protein members of the hnRNP family

**Figure 2:**
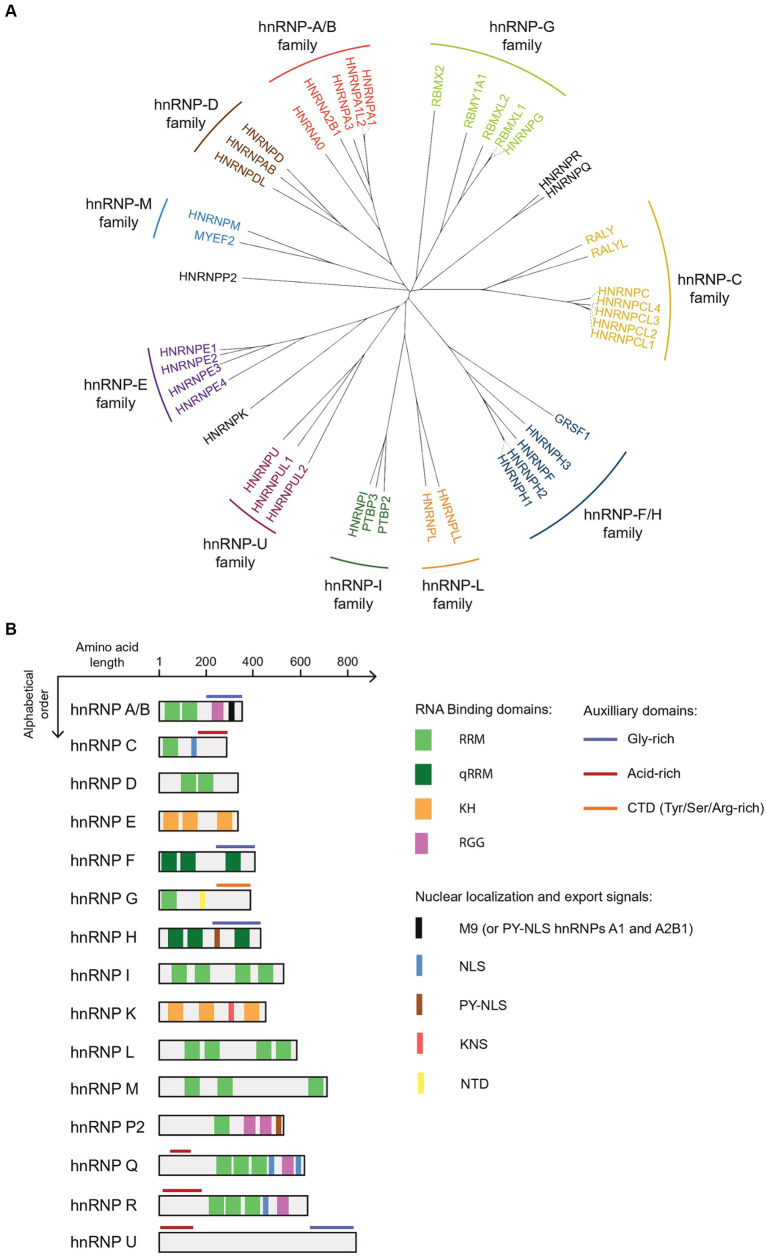
Structure and identity of members of hnRNP families. **(A)** Protein sequence comparison of hnRNPs by multiple sequence alignment program, Clustal Omega (https://www.ebi.ac.uk/jdispatcher/msa/clustalo). The protein sequences of the hnRNPs used correspond, for each member, to the highest expressed hnRNP isoform in the human cerebral cortex, identified via a ENST reference number (Ensembl Transcrit number) using the GTEx Transcript Browser program (https://www.gtexportal.org/home/transcriptPage) (Supplementary Table S1). The protein sequences corresponding to these isoforms were identified on the NCBI database from the ENST reference number (Supplementary Table S1). The percentage identity between members of hnRNP families can be found in Supplementary Table S2. **(B)** Schematic representation of the canonical structure for hnRNP sub-families. The schematic illustrates various domains: RRM (RNA recognition motif), qRRM (quasi RNA recognition motif), KH (K-homology domain), RGG (Arg-Gly-Gly repeat domain), NLS (Nuclear localization signal), PY-NLS (Proline/Tyrosine Nuclear localization signal), NTD (Nascent transcripts targeting domain), and KNS (hnRNP K nuclear shuttling).

The hnRNPs, which belong to the RNA-binding protein family, have been named after their initially identified role in packaging heterogeneous nuclear RNA (hnRNA) (Beyer et al., 1977). The classification of hnRNP members started with the recognition of the “core” hnRNP proteins (categorized into the A, B, and C groups) as major components of this family (Beyer et al., 1977). However, the wide range of molecular weights, spanning from 34 to 120 kDa (Choi and Dreyfuss, 1984) as well as similarities in the structure and sequence of hnRNPs with the same molecular weight have severely hampered identification of other members. Thanks to extensive sequence and structural analyses, hnRNP family is now defined as 20 canonical hnRNP sub-families designated from A (hnRNP A1) to U (hnRNP U) (Piñol-Roma et al., 1988), each of them being composed of several paralogues and in some cases, even distantly related proteins (Martinez-Contreras et al., 2007; Figure 2A). Yet, the classification of some hnRNP members is still under debate (Han et al., 2010b; Busch and Hertel, 2012; Geuens et al., 2016).

In the following sections, we will expand the discussion beyond the major hnRNPs initially identified by the Dreyfuss Lab (Dreyfuss et al., 1993). We will emphasize the emergence of hnRNP-like or minor members due to their conserved structure compared to canonical hnRNPs, but also highlight other mechanisms, such as alternative splicing and gene duplication, that further extend and add complexity to the exhaustive characterization of the hnRNP family.

### 2.2 Conserved structure across members of the hnRNP family

The analysis of the amino acid (aa) sequence of hnRNP members revealed multiple distinct RNA-binding domains (RBD), including RNA recognition motifs (RRM), quasi-RNA recognition motifs (qRRM), Arg-Gly-Gly repeat domain (RGG), or K homology domains (KH) (Dreyfuss et al., 1993), in all major members of the hnRNP family, except hnRNP U (Figure 2B). Close to the RBDs, hnRNP proteins also typically feature unstructured auxiliary domains with clusters rich in certain aa, such as acidic aa, glycine or proline (Geuens et al., 2016). Those auxiliary domains play dual roles in regulating protein–protein interactions and, in certain cases, subcellular localization. As example, the hnRNP A1 contains a nucleo-cytoplasmic shuttling (NS) domain named M9 within its auxiliary domain, characterized by its glycine-rich composition (Siomi and Dreyfuss, 1995). As such, hnRNPs show a modular composition arising from the combinations and arrangements of various domains, such as RBD and auxiliary domains, that increase their functional diversity (Han et al., 2010a).

The presence of several RBDs confer to hnRNPs the ability to bind multiple RNA sequences simultaneously (Singh and Valcárcel, 2005). Moreover, in addition to their binding to RNA, hnRNPs are concurrently engaged in protein–protein interactions. The interactions of hnRNPs with both proteins and RNA partners/targets are facilitated by their RBDs but also likely by low complexity domains (LCDs) within intrinsically disordered regions (IDRs) (Calabretta and Richard, 2015) or auxiliary domains (Biamonti and Riva, 1994; Cartegni et al., 1996).

As such, the model depicting the assembly of hnRNP G (RBMX) on exon 7 of SMN2 pre-mRNA showed that hnRNP G (RBMX) binds to RNA directly through its N-terminal RRM, and indirectly via the interaction of its C-terminal LCD with the splicing factor Tra2-β1 (Moursy et al., 2014). More recently, Van Lindt and collaborators demonstrated that hnRNP A2 interacts with various RNA molecules through a Try/Gly-rich motif located in the middle of IDR (Van Lindt et al., 2022), as previously suggested for the IDR LCDs of hnRNP A1 that is 72% identical to the IDR of hnRNP A2 (Abdul-Manan et al., 1996). Of note, LCDs present in hnRNPs are thought to participate in liquid–liquid phase separation resulting in the formation of membraneless organelles like nuclear speckles, processing bodies, and stress granules (discussed section 3.1).

Given the conserved RBD structure observed across various hnRNPs, RBPs with such domains have been proposed as members of the hnRNP family. Accordingly, the extensively studied TAR DNA binding protein 43 (TDP-43) is often associated to the hnRNP family and is well documented as a protein partner of many other hnRNPs (D'Ambrogio et al., 2009). On the same line, Raver1, that displays three RRM and that forms complexes with other hnRNP proteins, has also been qualified as a multidomain hnRNP-like protein. Further investigations have revealed, based on sequence similarities within RRM and their general domain organization, that *Raver1* has a related gene called *Raver2*, therefore classified as new member of the hnRNP family (Hüttelmaier et al., 2001; Kleinhenz et al., 2005). Two other RBPs, Msi2 and Msi1, are considered as members of the hnRNP family due to their structurally conserved sequences with hnRNP A/B and hnRNP D (AUF1), which are notably characterized by the presence of two copies of RRMs and one auxiliary domain (Sakakibara et al., 2001). Finally, a RBP known as cold-inducible RNA-binding protein (CIRBP) (Nishiyama et al., 1997), initially recognized for its role in response to cold stress, is also referred to as hnRNP A18 due to high sequence homology with members of the hnRNP family (Sheikh et al., 1997). Indeed, the human hnRNP A18 (CIRBP) comprises a structured N-terminal domain with an RRM, and a C-terminal low-complexity region containing the RGG and RSY regions (Bourgeois et al., 2020).

As RBDs, IDRs, or LCDs can also bind single-stranded DNA (ssDNA) (Dettori et al., 2021), hnRNPs have been thought to interact with DNA. As such hnRNP E1 (PCBP1) and members of the hnRNP A/B family, including hnRNPs A1, A2/B1, and A3, have been documented to associate with single-stranded telomeric DNA and therefore participate in telomere biogenesis (McKay and Cooke, 1992; LaBranche et al., 1998; Ding et al., 1999; Moran-Jones et al., 2005; Tanaka et al., 2007; Mohanty et al., 2021). *In vitro* experiments have demonstrated that hnRNP U can bind ssDNA through its C-terminal glycine-rich region (Kiledjian and Dreyfuss, 1992). A binding affinity test of TDP-43 has also revealed an interaction with single-stranded DNA fragments derived from the HIV-1 TAR sequences (Kuo et al., 2009). Other evidence comes from hnRNP G (RBMX) that is recruited, in response to replication stress, to repetitive DNA sites where it activates the genome surveillance pathway (Zheng et al., 2020). This function is independent of hnRNP G (RBMX) interaction with nascent RNA but involved a poorly characterized RBD, termed RBM1CTR and located within the middle of the hnRNP G (RBMX) protein (Zheng et al., 2020). Of note, like hnRNP G (RBMX) (Adamson et al., 2012), the RRM of many other hnRNPs, including hnRNP R (Ghanawi et al., 2021), hnRNP U (Britton et al., 2014), hnRNP P2 (FUS) (Mamontova et al., 2023) and hnRNP D (Alfano et al., 2019), mediates their recruitment to ssDNA sites upon DNA damage to ultimately facilitate DNA damage response. These roles do not always require a direct binding to DNA but are rather dependent of β-H2AX or PARP1 proteins, that are known to mediate the recruitment of repair proteins to the DNA lesion. Furthermore, the knockdown of hnRNP K leads to DNA repair defects and initiates a DNA damage response (DDR) upon gamma irradiation. This process is facilitated by the upregulation of DDR genes such as *p21* and *p53* (Wiesmann et al., 2017). Although there is increasing evidence for roles of hnRNPs in the regulation of genome stability, as highlighted in recent reviews (Klaric et al., 2021; Provasek et al., 2022), we will focus, in the next section of this review, on their function within the spliceosome.

### 2.3 Factors contributing to the large membership of the hnRNP family

#### 2.3.1 Alternative splicing of *hnRNP* transcripts

Transcripts encoding major hnRNPs are themselves subjected to alternative splicing (Ezkurdia et al., 2012). It emerges that: (1) nearly all hnRNP members exhibit various isoforms, (2) one isoform frequently appears dominant in expression, and (3) different isoforms are expressed depending on the tissue (see section 3). The *hnRNP I* gene (also known as *PTBP1*) comprises 15 exons. Exon 9 undergoes alternative splicing, leading to the generation of multiple isoforms (Romanelli et al., 2000). The exclusion of exon 9 decreases the inhibitory function of hnRNP I (PTBP1) and enables the initiation of a specialized alternative splicing program specific to the brain (Gueroussov et al., 2015). The *hnRNP R* gene is also subjected to alternative splicing, resulting in the production of two unique protein isoforms, hnRNP R1 and hnRNP R2. hnRNP R1 comprises 633 aa, whereas hnRNP R2 lacks 38 aa distributed across its acidic domain and RRM. The expression patterns of hnRNP R1 and hnRNR R2 vary significantly depending on the tissue. While hnRNP R1 exhibits ubiquitous expression and significantly higher levels compared to hnRNP R2, the latter shows low expression levels specifically in neural tissue (Huang et al., 2005; Cappelli et al., 2018). hnRNP Q exhibits close structural similarities to hnRNP R and undergoes alternative splicing, resulting in three isoforms of hnRNP Q denoted as Q1–Q3 (Mourelatos et al., 2001). Several alternatively spliced hnRNP E2 (PCBP2) mRNAs exist, with the full transcript isoform serving as a model for the retrotransposition event that gave rise to the *hnRNP E1* (*PCBP1*) intronless gene (Makeyev et al., 1999). The principal constituents of the hnRNP A family (hnRNP A1, hnRNP A2/B1, hnRNP A3) are also alternative spliced. hnRNP A1 produces transcripts A1 and A1b, hnRNP A2/B1 is spliced into transcripts B1, A2, A2b, and B1b, and hnRNP A3 generates transcripts A3a and A3b (Han et al., 2010a). Interestingly, a tissue-specific expression patterns of hnRNP A3 isoforms were observed in mice. hnRNP A3b is the predominant isoform in all assessed rodent tissues, except in the brain, where the unspliced A3a isoform exhibited significant overexpression (Han et al., 2010a; Papadopoulou et al., 2012). hnRNP D encompasses four isoforms (p45, p42, p40, and p37) with common structural elements generated through alternative splicing of a shared pre-mRNA. The p42 and p45 isoforms of hnRNP D are predominantly located in the nucleus, while the smaller variants (p40 and p37) are present in both the nuclear and cytoplasmic compartments (White et al., 2013). Strikingly, data of hnRNPs expression that can be found in the Genotype-Tissue Expression (GTEx) portal confirmed that nearly all *hnRNP* genes express multiple isoforms. For those that do not, this phenomenon is attributed to intronless *hnRNP* genes, such as *hnRNP E1* (*PCBP1*) or *RBMXL1*, or to genes that are not expressed in this tissue, such as *hnRNP CL1-4*. Finally, it has been shown that the alternative splicing of the exon 2 of the hnRNP R transcript results in an isoform with a truncated N-terminus, that loses its interaction with Yb1 and the associated function in DNA damage repair (Ghanawi et al., 2021).

Collectively, it appears that alternative splicing largely contributes to the diversity of hnRNPs, by leading to specific expression patterns and/or modifying functions through removal or partial alteration of some functional domains in the spliced isoforms. Thanks to the emergence of the long-read sequencing, we foresee the discovery of many other hnRNP isoforms. As a proof of principle, such technology has revealed a previously uncharacterized isoform of hnRNP A18 (CIRBP) and a shift from the canonical CIRBP-201 isoform to the new CIRBP-210 isoform in infected epithelial cells (Corre et al., 2023).

#### 2.3.2 Evolution: gene duplication and retrotransposition events

The fact that the number of families and number of members within a given family expanded with the emergence of more complex multicellular organism suggests: (i) that hnRNPs have evolved from a common ancestor gene mainly through gene duplication (Busch and Hertel, 2012); and (ii) the presence of strong selective pressures acting on duplicated hnRNP genes (Busch and Hertel, 2012). The increase in the number of major hnRNPs as well as the emergence of additional members that could be designated as minor hnRNP members have been also attributed to retrotransposition events (Chaudhury et al., 2010). Retrocopied genes originate from insertion of retro-transcribed mRNA into the genome. As such, retrocopies lack introns and regulatory sequences found in their parent genes and are often non-functional or qualified as “processed pseudogenes” (Hatfield et al., 2002). However, in some cases, retrocopies may acquire novel functions through the acquisition of mutations or regulatory elements present in their genomic location, thereby contributing to genetic diversity and evolution (Seczynska and Lehner, 2023).

A striking illustration of the intricate evolutionary processes entailing gene duplication and retrotransposition events is exemplified by *RBMX*, encoding hnRNP G on the X chromosome. hnRNP G is part of the hnRNP sub-family with the highest number of paralogs (duplicated genes within the same species). These paralogs comprise duplicated genes with similar intron/exon organizations located on the Y chromosome, originating from an ancestral pair of X/Y chromosomes (Lingenfelter et al., 2001; Elliott et al., 2019). In humans, the long arm of chromosome Y harbors six functional, nearly identical copies (RBMY1A1, RBMY1B, RBMY1D, RBMY1E, RBMY1F, and RBMY1J), along with over 20 pseudogenes (Elliott et al., 2000; Elliott, 2004). Unlike hnRNP G (RBMX), which shows ubiquitous expression, *RBMY* genes display a specific expression pattern, primarily in the testes (Elliott et al., 1998). Yet, they are both involved in Tra2β-dependent pre-mRNA splicing (Venables et al., 2000). In addition to the duplicated genes on chromosome Y, *hnRNP* *G* (*RBMX*) has undergone multiple retrocopies throughout evolution, resulting in at least nine intronless copies present in the human genome (Lingenfelter et al., 2001; Elliott et al., 2019). Some of the earliest gene duplications and retrocopies, such as the *RBMXP1-5* pseudogenes (Stelzer et al., 2016), are nonfunctional, while others have retained functionality but have adopted new expression patterns, as seen for *RBMXL2* and *RBMXL9* whose expression is restricted to the testes and brain (Lingenfelter et al., 2001; Ehrmann et al., 2008). In humans, the most recent retrocopy located on chromosome 1 (*RBMXL1*) is ubiquitously expressed and encodes a protein that shares 96% identity with hnRNP G (RBMX) (Figure 2A; Lingenfelter et al., 2001).

Various examples of gene duplication and retrotransposition events can be also found in other hnRNP sub-families: (1) Like *hnRNP G* (*RBMX*), *hnRNP E* has 3 paralogues that arose from two duplication events. Interestingly, one of those, *PCBP2* (*hnRNP E2*), has been subjected to two evolutionary independent retrotransposition events, generating 3 retrocopies, *PCBP1* (*hnRNP E1*), *PCBP2P1* and *PCBP2P2* (Makeyev et al., 1999; Makeyev and Liebhaber, 2000). (2) Comparison of the domain architecture of the hnRNP A/B family members revealed that hnRNP A1 and hnRNP A2, that exhibit a 68% aa identity (Biamonti et al., 1994; Mayeda et al., 1994), arose from the duplication of a common ancestral gene, rather than from an independent assembly of domains (Biamonti et al., 1994). Further investigation into this sub-family has revealed in mice that the *hnRNP A2* (that gives rise to four isoforms, A2, B0a, B0b, and B1) and *hnRNP A3* genes have 5 and 7 (14 in humans) processed pseudogenes, respectively, most of them being non-functional (Hatfield et al., 2002; Makeyev et al., 2005), except one *hnRNP A2* pseudogene that contains putative promoter sequences and may potentially produce a functional protein (Hatfield et al., 2002). (3) One isoform of *hnRNP I* (*PTBP1*), known as *PTBP3* has been retrotranscribed and inserted in the genome to give rise to the *ψhnRNP I* pseudogene whose activity remains uncertain (Romanelli et al., 2000). (4) Four processed pseudogenes have been identified in the hnRNP K sub-family, though none of them seem to be functional (Leopoldino et al., 2007).

**Figure 3:**
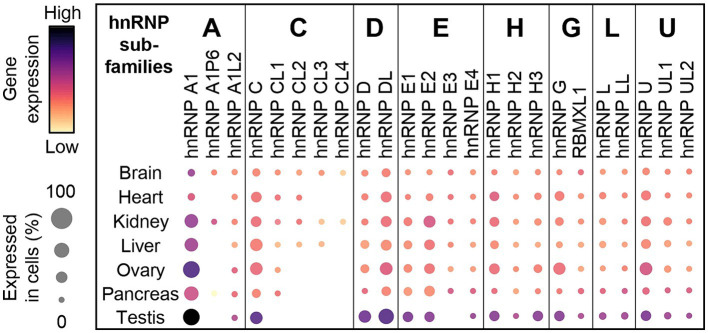
Gene expression of the parental hnRNP members, along with their duplicated and retrotransposed genes within the sub-family across multiple organs. Expression profiles of hnRNP sub-family members (A, C, D, E, H, G, L, and U) across various human organs, including the brain, heart, kidney, liver, ovary, pancreas, and testis, obtained from CZ CellxGene Discover platform and showing higher expression of the parental genes. The dot plot was made using the gene expression normalized as described in the CZ CellxGene Discover platform (https://cellxgene.cziscience.com/).

Although duplication and retrotransposition events clearly participate to the expansion of the number of hnRNPs throughout evolution, it is puzzling that only few paralogues have been shown to be functional. Also, strong sequence homology (Figure 2A) raises the possibility of redundant function among paralogues (discussed in section 5). Interestingly, expression of most duplicated genes and retrocopies is several folds lower than the parent gene (Figure 3), suggesting that processed pseudogenes might become critical in specific context, in particular when the parent gene is not expressed.

Finally, comparison of the sequences of the highest expressed hnRNP isoforms in the cerebral cortex question the need of revising the classification of hnRNPs, notably in sub-families. For instance, hnRNP R and hnRNP Q that share 82% of sequence homology belong to two distinct families, while RALY and RALYL that show up to 47% of similarities with hnRNP C are considered as members of the hnRNP C family (Figure 2A). In addition, the minimum homology within a family ranges from 43% (hnRNP C) to up to 90% [hnRNPs C and G (RBMX)] (Figure 2A), suggesting that the homology and organization of specific domains might preferentially account to define the members of a family.

We anticipate the number of hnRNPs to greatly increase in the future thanks to the advent of next-generation sequencing, advanced bioinformatics analyses and structural methods, which would be determinant to identify genes resulting from duplication or retrotransposition events (Vollger et al., 2019; Feng and Li, 2021).

#### 2.3.3 The relationship between viruses, retroviruses and hnRNPs: causes of retrotransposition events?

The hnRNP families are involved in various steps of the viral life cycle, including biosynthesis (i.e., RNA synthesis, RNA translation) and release stages (Wang et al., 2022). For their replication and propagation, viruses rely on host molecular components such as splicing factors like hnRNP proteins and SR proteins (Bolinger and Boris-Lawrie, 2009; Boudreault et al., 2019). In accordance, it has been shown that the expression of hnRNPs are modified following viral infection. Interestingly, hnRNPs could be either upregulated or downregulated upon a same viral infection. As an example, during HIV-1 infection, hnRNP A1 expression is increased (Monette et al., 2009) while hnRNPs A2/B1 and H are decreased (Dowling et al., 2008). Conversely, expression of a given hnRNP could be either increase or decrease depending on the nature of the virus as shown for hnRNP A1 that is upregulated during influenza A virus (IAV), HIV-1 (Monette et al., 2009), and HPV16 (Cheunim et al., 2008) infections, and downregulated during infections with porcine epidemic diarrhea virus or snakehead vesiculovirus (Li et al., 2018; Kaur et al., 2022; Liu et al., 2023). Moreover many hnRNPs [hnRNP D (Lund et al., 2012), hnRNP A1, hnRNP K, hnRNP C1/C2 (Pettit Kneller et al., 2009), hnRNP K (Burnham et al., 2007; Brunetti et al., 2015), hnRNP H (Redondo et al., 2015), and hnRNP M (Jagdeo et al., 2015)] have been shown to relocalize to the cytoplasm following infection with various viruses. In the cytoplasm, those hnRNPs likely interact with viroplasmic proteins NSP2 and NSP5 that serve as the primary site for viral replication and assembly (Dhillon et al., 2018).

Several hnRNPs have also been shown to modulate the viral propagation within infected cells. As such, hnRNP G (RBMX) has been shown to interact with HIV-1 long terminal repeat (LTR) promoter region, where it sustains repressive trimethylation of histone H3 lysine 9 (H3K9me3), prevents the recruitment of the RNA polymerase II and consequently inhibits HIV-1 transcription (Ma et al., 2020). Conversely, hnRNP A2/B1 interacts with LTR G-quadruplexes, functioning as an activator of HIV-1 transcription (Scalabrin et al., 2017). hnRNP H1 is crucial for HIV-1 replication, as it binds to purine-rich sequences on the viral RNA. Depletion or mutation of its binding sites leads to decreased expression of Vif protein, hindering viral replication efficiency (Kutluay et al., 2019). Furthermore, many hnRNPs have been demonstrated to regulate HIV-1 Gag expression: (i) the four isoforms of hnRNP D exert distinct effects on HIV-1 Gag expression, with the longest isoforms, p45 and p42, enhancing viral Gag synthesis, and the shorter isoforms, p40 and p37, inhibiting it (Lund et al., 2012); (ii) hnRNP E1 (PCBP1) reduces cap-dependent translation initiation of HIV-1 viral RNA, resulting in decreased Gag synthesis (Woolaway et al., 2007); (iii) 21 hnRNPs have been identified in at least on affinity purification/mass spectrometry screenings that aimed at discovering potential cellular interaction partners of HIV-1 Gag (Engeland et al., 2014). Interestingly, 6 of them were also shown to bind the HIV-1 5’ UTR (Stake et al., 2015).

Altogether, there data indicate that, on one side, hnRNPs are hijacked by viruses for their replication in the host cells, and on the other side, this class of protein is very prone to duplication and retrotransposition events, raising the possibility that those events are correlated.

## 3 Localization, expression, and regulation

### 3.1 Intracellular localization

Consistent with their well-described role in splicing, the majority of hnRNP proteins are found in the nucleus under physiological conditions (Piñol-Roma, 1997). For that matter, hnRNPs represent one of the most abundant family of proteins in the nucleus (Dreyfuss et al., 2002). Nuclear localization of hnRNPs is mediated by classical nuclear localization sequence (NLS) as well as non-classical PY-NLS (proline-tyrosine NLS, also known as M9 domain) (Piñol-Roma, 1997; Purice and Taylor, 2018; Khalil et al., 2024). Although, hnRNP proteins have been long thought to be excluded from the nucleolus, proteomic analysis of the human nucleolus revealed that hnRNPs A1, A3, A2/B1, C, G (RBMX), H1, H3, and K are components of the nucleolar proteome (Andersen et al., 2002). Strikingly nucleolar association of some of them [hnRNPs K, G (RBMX), and A2/B1] is enhanced when transcription is inhibited (Andersen et al., 2002). In addition, one recent study used immunofluorescence to show a colocalization of hnRNP UL1 with nucleolin, the major nucleolar protein, in HeLa cells (Cichocka et al., 2022). However, it is worth mentioning that immunogold electron or immunofluorescence microscopy did not show any labeling of hnRNP C and A2/B1 in the nucleolus of human cells (Romero et al., 1998; Friend et al., 2008), the discrepancy with the proteomic data likely coming from the different sensitivity in the methods used. On the same line, tagged version of hnRNP G (RBMX) (Matsunaga et al., 2012) or hnRNP P2 (FUS) (Yang et al., 2014) are not found in the nucleolus after overexpression. Although one can argue that tagging or overexpression of hnRNPs hamper their nucleolar localization, it is puzzling that hnRNP G tagged-proteins that lack its tyrosine-rich region (TRR) but not the ones that lack the RRM, localized to the nucleolus (Matsunaga et al., 2012). Finally, one might anticipate that the advent of highly sensitive proteomic methods will increase, in the future, the number of hnRNPs associated to the nucleolus.

As shown for pre-mRNA splicing factors, several hnRNPs have been found in nuclear speckles. The monoclonal antibody SC35, frequently used to mark nuclear speckles, has been used to perform immunoprecipitation coupled with mass spectrometry on the leukemia human HAP1 cell line. The results revealed numerous hnRNPs as interactants, including hnRNP M, hnRNP C, hnRNP K, hnRNP G (RBMX), and hnRNP U in the top 50 hits (Ilik et al., 2020). A study also reported several hnRNPs [A1, A1L, F, G (RBMX), H1, H3, K, R, and UL1] as key components of paraspeckles (Naganuma et al., 2012), that are typically located in close proximity to nuclear speckles and enriched in specific long non-coding RNAs and RBPs.

It has long been established through pioneering research that certain hnRNP proteins exhibit continuous shuttling between the nucleus and cytoplasm, rather than remaining exclusively within the nucleus (Piñol-Roma and Dreyfuss, 1992). One of the first hnRNP proteins described to undergo nucleocytoplasmic shuttling is hnRNP A1 (Weighardt et al., 1995), through its M9 domain (also known as the PY-NLS) (Izaurralde et al., 1997; Soniat and Chook, 2016). This has been then expanded to other hnRNPs (D, E, I, and K). However, some hnRNPs are exclusively retained in the nucleus such as hnRNPs C and U (Piñol-Roma, 1997) or hnRNP DL (Zhang et al., 2021). In the cytoplasm, hnRNPs can have opposite effects on mRNA stability, promoting either stabilization or degradation. Indeed, many hnRNPs can regulate either positively or negatively rapid mRNA decay. For instance, it has been shown that all the hnRNP D isoforms promote decay by binding to mRNA-destabilization sequence (Loflin et al., 1999; Xu et al., 2001; Fialcowitz et al., 2005). Likewise, hnRNP A2/B1 and hnRNP A1 have been demonstrated to initiate mRNA degradation by facilitating the recruitment of the CCR4-NOT deadenylase complex through their binding to UAASUUAU sequence in the mRNA 3′UTR (Geissler et al., 2016). hnRNPs can also cooperate with RBPs from different families to induce mRNA decay, as demonstrated for hnRNP F, that serves as a co-factor in TTP/BRF1-dependent mRNA degradation (Reznik et al., 2014). Interestingly, this role of hnRNP F is independent of its binding to the mRNA targeted for decay (Reznik et al., 2014). In contrast, hnRNP I (PTBP1) protects mRNAs from degradation by binding to their 3’ UTR and preventing the binding of the NMD helicase UPF1 to the 3’UTRs (Ge et al., 2016). Two other hnRNPs, hnRNP L, and hnRNP I (PCBP1), possess the capability to remove the UPF1 NMD factor from the 3’ UTR of particular mRNAs, including CFTR mRNA (Siddiqui et al., 2023), safeguarding these transcripts against NMD (Kishor et al., 2019). Interestingly, various hnRNPs can regulate differently the same mRNA. This is exemplified by the regulation of the mouse *Period3* (m*Per3*) mRNA, that is a binding target for several hnRNPs [D, K, I (PTBP1), and Q]: while hnRNP K preserves m*Per3* stability, hnRNPs D and Q promote its degradation and hnRNP I (PTBP1) show no impact on m*Per3* stability (Kim et al., 2011, 2015). Interestingly, hnRNPs Q, D, and I (PTBP1) as well as hnRNP R, also contribute to the oscillation of the circadian mRNAs *Per2*, *Cry1*, and *Nat* (Kim et al., 2005; Woo et al., 2009, 2010). Increase stabilization of mRNA has been also demonstrated for several hnRNPs, although the underlying mechanisms have not been clearly elucidated yet: (i) the stability of *APP* mRNA can be increased by the binding of hnRNPs (F, H1, and C) (Rajagopalan et al., 1998; Khan et al., 2021); (ii) the interaction between hnRNP H/F and the G-quadruplex located at the 3′ end of *p53* mRNA reinforces the binding of hnRNP H/F to *p53* mRNA, increasing its expression in response to DNA damage (Decorsière et al., 2011); (iii) hnRNP L binds and stabilizes the *BCL2* mRNA, which plays a critical role in regulating apoptosis (Lim et al., 2010); (iv) hnRNP U has been demonstrated to modulate the expression of TNFα and several other mRNAs (*GADD45A, HEXIM1, HOXA2, IER3, NHLH2*, and *ZFY*) by promoting mRNA stability (Yugami et al., 2007); (v) hnRNPs E1 (PCBP1) and E2 (PCBP2) regulate the stability of the androgen receptor mRNA (Yeap et al., 2002). Several other cases of hnRNP E1’s role in the regulation of mRNA stability have been reviewed by Chaudhury et al. (2010); (vi) hnRNP E1 (PCBP1) controls *p63* mRNA stability by binding to its 3’UTR, particularly the CU-rich element (Cho et al., 2013).

Another important cytoplasmic function resides in the control of translation. First, it has been shown that hnRNP E1 (PCBP1) promotes translation by interaction with the IRES of some mRNAs (Gamarnik and Andino, 1997; Evans et al., 2003; Pickering et al., 2003). This function is shared with many other hnRNP members, as described in this review by Godet et al. which extensively analyzes IRES trans-acting factors (ITAFs) regulating cellular IRESs. Among ITAFs, one can find nuclear proteins capable of shuttling between the nucleus and cytoplasm to govern IRES-dependent translation, including hnRNPs (A1, C, D, E, H2, I, K, L, M, Q, and R) (Godet et al., 2019). Second, several evidence highlight the association of several hnRNPs with ribosomes: (i) hnRNP C, hnRNP G (RBMX), hnRNP H3, and RALY have been found enriched in polysomes fraction during mitosis (Aviner et al., 2017); (ii) other hnRNPs [such as hnRNP E1 (PCBP1), hnRNP E2 (PCBP2), hnRNP A2/B1, and hnRNP I (PTBP1)] have been shown to be associated with polysome under hypoxic conditions, while others showed either no change (hnRNP A3) or reduced translational engagement (hnRNP C) (Ho et al., 2020); and (iii) hnRNP M distribution shifts from monosome fractions under normoxia to polysome fractions under hypoxia, suggesting increased translation activity in response to low oxygen levels (Chen et al., 2019).

Cytoplasmic hnRNPs can also orchestrate the transport of mRNA molecules to precise locations, notably in axons. A recent study has shown that PTBP2 binds and facilitates the trafficking of *hnRNP R* mRNA into axons, consequently enabling the local synthesis of hnRNP R within axons (Salehi et al., 2023). Interestingly, hnRNP R itself may play a role in the axonal translocation of *β-actin* mRNA (Glinka et al., 2010) or of the non-coding RNA 7SK (Glinka et al., 2010; Briese et al., 2018), functions that both sustain axonal growth (Glinka et al., 2010; Briese et al., 2018). RNA co-immunoprecipitation (RIP) with axonal hnRNPs further revealed that various hnRNP proteins [AB, A1, A2/B1, A3, D, DL, E2 (PCBP2), E3, F, H1, H2, I, PTBP2, PTBP3, K, L, R, and U] work together to regulate mRNA transport within axons through their binding to specific mRNA motifs (Lee et al., 2018). Strikingly, axotomy increased the axonal transport of RNA granules containing hnRNPs (H1, F, and K), which exhibit a preference for binding to mRNAs essential for axon regeneration (*nrn1* and *hmgb1*) (Lee et al., 2018). On the same line, hnRNP A/B interacts with mRNAs encoding proteins involved in axon projection and synapse assembly, thereby promoting their local translation and accurate expression of the encoded protein at axon terminals in olfactory sensory neurons (Fukuda et al., 2023). Although it becomes clear that hnRNPs could promote the trafficking of some mRNAs, whether hnRNPs bind fully mature mRNAs or whether those are spliced or processed while transported remain to be determined.

Cytoplasmic hnRNPs are also involved in the formation of membraneless organelles, such as stress granules (SGs). This function is conferred by their LCDs, which facilitate liquid–liquid phase separation mechanisms responsible for the formation of these SGs (Purice and Taylor, 2018). For instance, the LCD of hnRNP A1 induces liquid–liquid phase separation (LLPS) *in vitro* and is sufficient for recruitment into SGs in cells. Notably, elevating the cytoplasmic concentration of hnRNP A1 and closely related RBPs is enough to trigger SG formation, supporting the idea of an LLPS-mediated mechanism (Molliex et al., 2015). Interestingly, some hnRNPs, including hnRNP A1, hnRNP K and hnRNP H relocalize, under cellular stress conditions, to the cytoplasm, where they accumulate in SGs, playing a crucial role in the cellular stress recovery (Guil et al., 2006; Fukuda et al., 2009; Wall et al., 2020). SGs are hallmarks of neurodegenerative disorders, particularly amyotrophic lateral sclerosis (ALS) and frontotemporal dementia (FTD) (Dudman and Qi, 2020; Nedelsky and Taylor, 2022). Interestingly, in an effort to characterize the protein composition of SGs, a comprehensive analysis of the available dataset by Asadi et al. revealed that many hnRNPs localized to SGs in various neurodegenerative conditions including the ALS/FTD continuum, Alzheimer’s disease (AD), Multiple sclerosis (MS) and Motor neuron disease (MND): TDP-43 and hnRNP P2 (FUS) are found in many if not all pathological conditions, while hnRNP A2/B1 and hnRNP A0 are associated to SG in ALS and AD, respectively (Asadi et al., 2021). Through bioinformatics analyses, hnRNP C, hnRNP DL, hnRNP H1, hnRNP F, hnRNP A2/B1, and hnRNP I (PTBP1) were also predicted to interact with SGs (Asadi et al., 2021).

Altogether, these findings suggest that nucleo-cytoplasmic shuttling of hnRNPs might play critical role in regulating the localization and/or translation of numerous mRNAs in both physiological and pathological contexts.

### 3.2 hnRNPs expression in the brain

**Figure 4:**
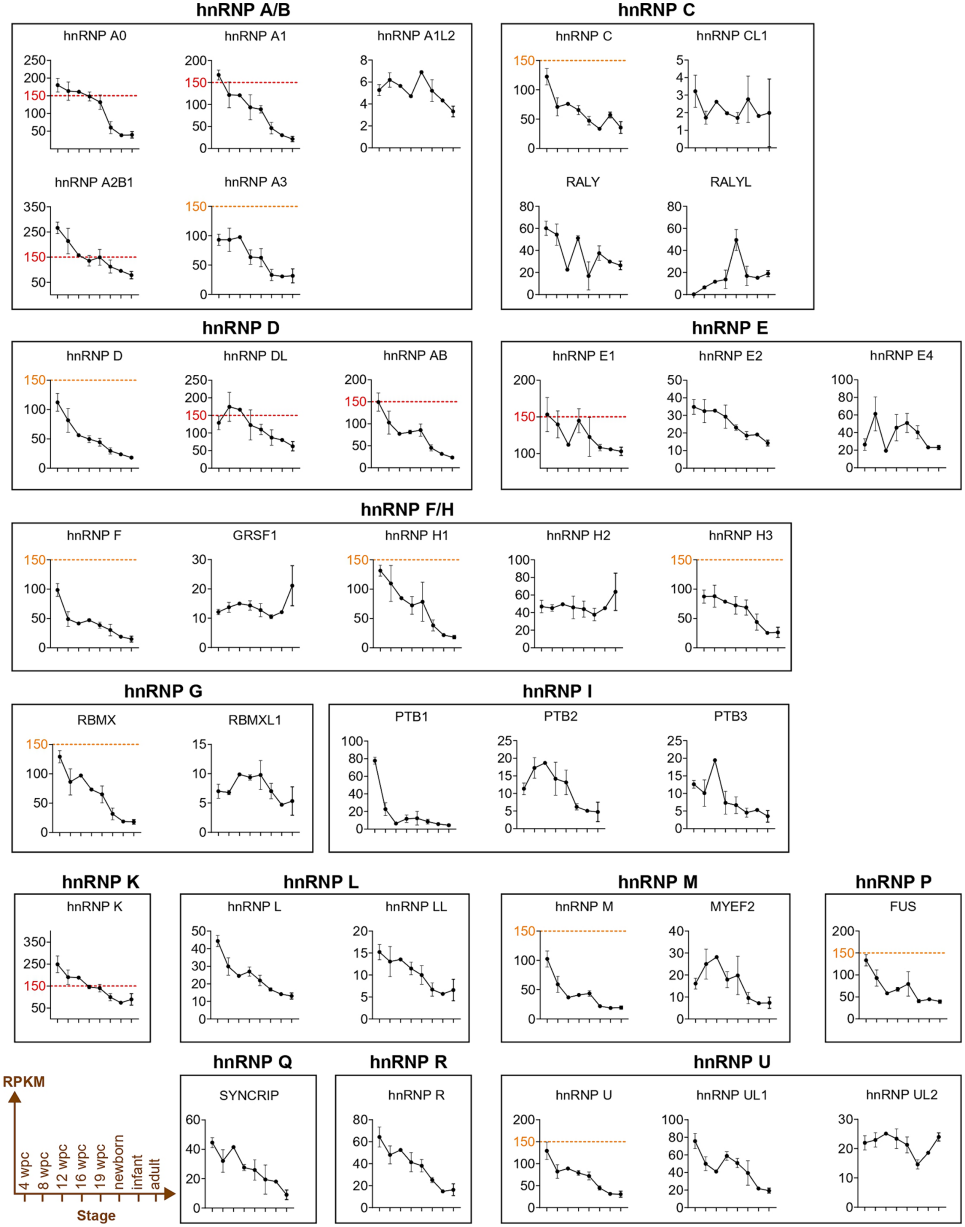
The gene expression profiles of hnRNP proteins in the human brain across various developmental stages. Overview of gene expression profiles of the hnRNPs members across human brain from a selection of developmental stages [4-, 8-, 12-, 16-, or 19-wpc (weeks post-conception)], newborn, infant, and young adults (25–32 years) using the resource provided by the Kaessmann Lab (Cardoso-Moreira et al., 2019). Expression levels were calculated in million mapped reads per kilobase of exon (RPKM). The red dashed line corresponds to hnRNPs that reach at least 150 RPKM. The orange dashed line refers to hnRNPs that are close to 150 RPKM. No expression was detected for hnRNP A1L1 (also known as hnRNP A1P6), hnRNP CL2, hnRNP CL3, and hnRNP E3.

The initial observations came from Dreyfuss and colleagues, who reported a higher expression of several hnRNP proteins (A1, C, D, F/H, K/J, L, and U) in the brain, ovary, and testis compared to other organs (Kamma et al., 1995). More specifically, they also demonstrated, using immunostaining, that neurons exhibit significantly stronger staining intensity than glial cells for all hnRNP proteins. In particular, cerebellar Purkinje cells and large ganglion cells of the basal ganglia expressed more hnRNP proteins than small neuronal cells or glial cells (Kamma et al., 1995, 1999). The high expression of hnRNP proteins in brain tissues, that correlates with the fact that alternative splicing occurs at the highest frequency in the brain (Mazin et al., 2021), has been confirmed by genome-wide transcriptomic analyses performed in seven different organs (brain, cerebellum, heart, kidney, liver, ovary, and testis) at various developmental stages spanning from early organogenesis to adulthood in humans (Cardoso-Moreira et al., 2019).^3^ Interestingly, in all organs including brain, the expression of *hnRNP* transcripts strikingly decreases during the perinatal period (Cardoso-Moreira et al., 2019; Figure 4). These transcriptomics data, along with the GTEX data reanalysis by Gillentine et al. (2021) confirmed that hnRNPs A0, A1, A2/B1, DL, E1 (PCBP1), K, G (RBMX), U, and P2 (FUS) are the most highly expressed hnRNPs in all brain regions analyzed (Figure 4, ≥150RPKM), with a decrease in expression observed during the brain development for almost all of them, except for RALYL, GRSF1, hnRNP H2, and hnRNP UL2 (Cardoso-Moreira et al., 2019; Figure 4). Notably, cerebellum tends to show a higher expression of all hnRNP members (Gillentine et al., 2021). An important point is the different pattern of expression observed between the members of a same hnRNP family, as shown for hnRNP C, hnRNP I (PTBP1), hnRNP F/H, and U (Cardoso-Moreira et al., 2019; Figure 4). Collectively, hnRNP expression in the human brain is subject to spatial and temporal regulation. The spatiotemporal regulation of hnRNPs is illustrated by: (i) PTBP1 (hnRNP I) and PTBP2 (nPTB), whose expressions are almost mutually exclusive. During brain development, cells switch from expressing hnRNP I (PTBP1) to PTBP2, thereby contributing to the neuronal differentiation process (Boutz et al., 2007), and (ii) hnRNP A1 and one of its isoforms, hnRNP A1B show different expression pattern and subcellular localization with hnRNP A1B more restricted to the central nervous system and found in neuronal processes compared to hnRNP A1 (Gagné et al., 2021).

**Figure 5:**
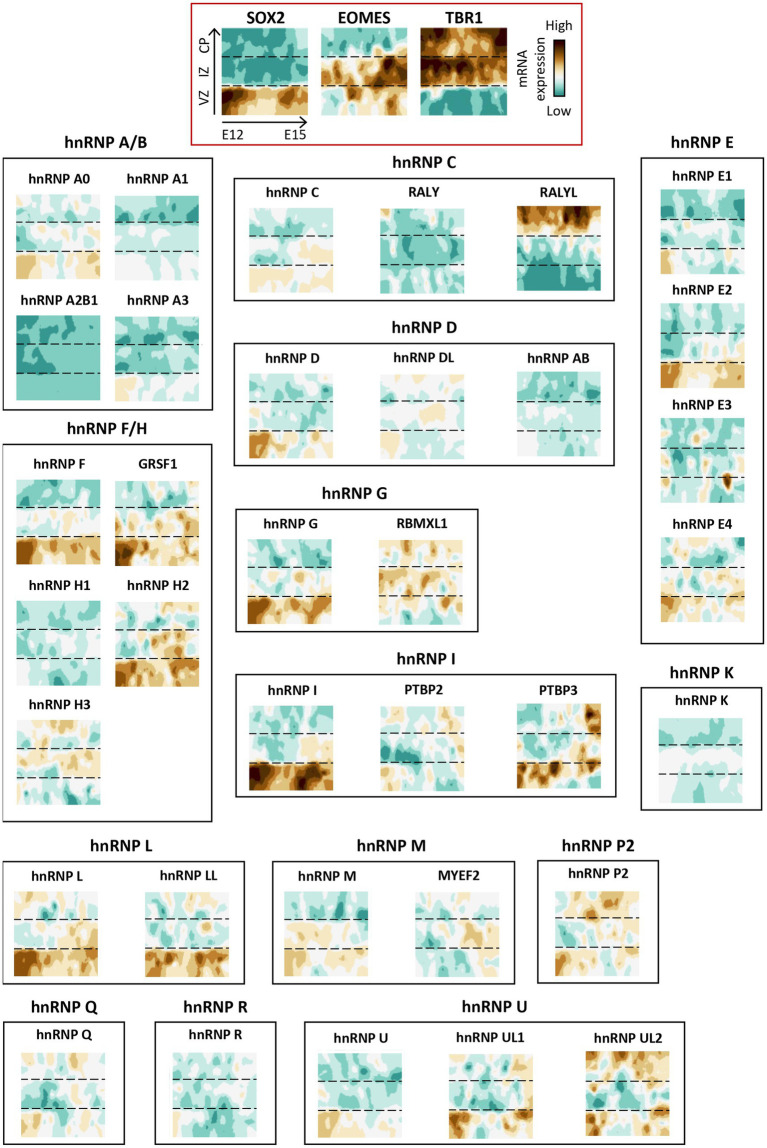
Spatio-temporal expression of hnRNP members during corticogenesis in mice. Spatio-temporal expression of hnRNP members from a single-cell RNAseq analysis in mouse developing cortices (Telley et al., 2019). The data were obtained from the open website (http://genebrowser.unige.ch/telagirdon/). X axis is time of apical progenitor birth, Y axis represents time of neuron differentiation. SOX2, EOMES (TBR2), and TBR1 have been utilized as markers to delineate the ventricular zone (VZ) progenitor, newly generated neurons in intermediate zone (IZ), and post-migratory neurons in the cortical plate (CP) respectively.

**Figure 6:**
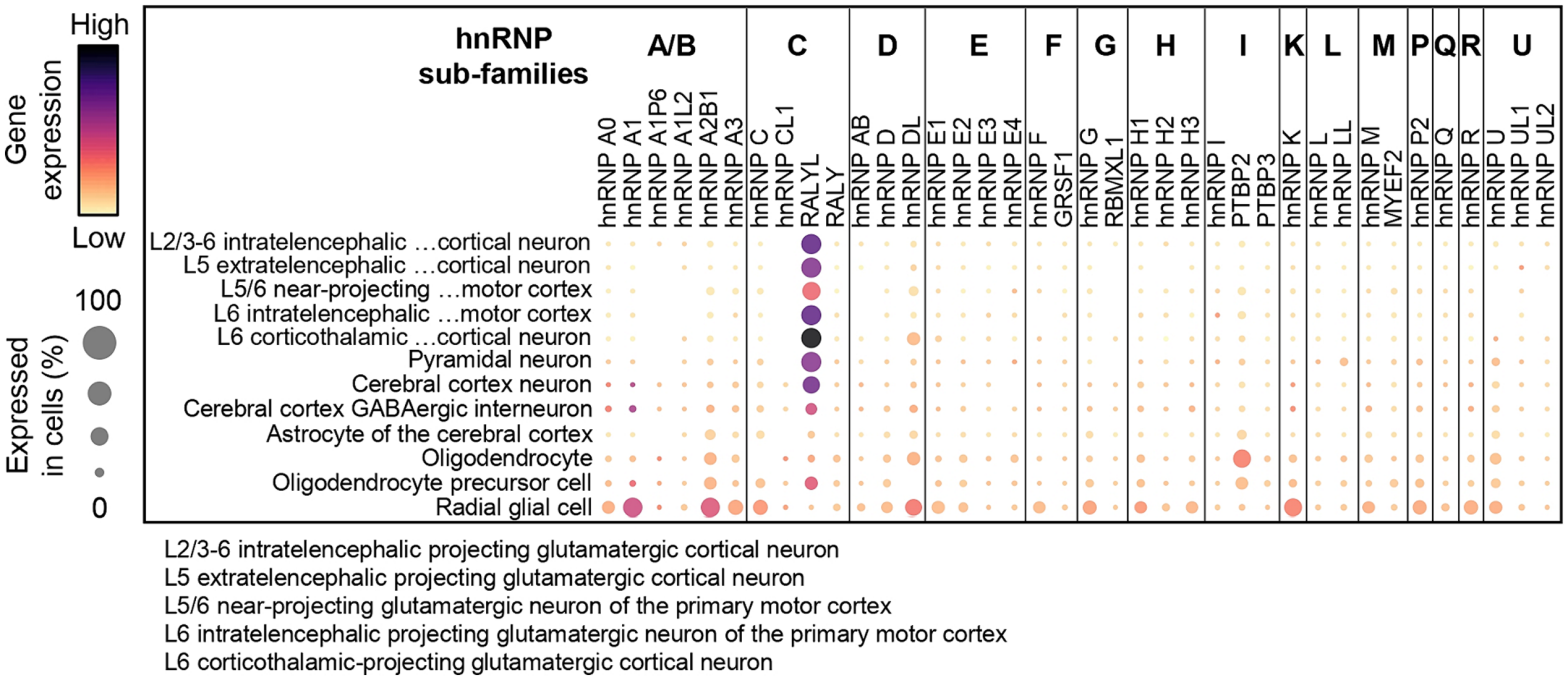
hnRNP expression patterns across various neuronal cell types in the human brain. Expression profiles of hnRNP sub-families in cortical progenitors (radial glial cells), cortical neurons (excitatory neurons from different cortical layers, inhibitory interneurons) and glia cells (astrocytes, oligodendrocytes, oligodendrocyte precursors) in the human brain. The dot plot was made using the gene expression normalized as described in the CZ CellxGene Discover platform (https://cellxgene.cziscience.com/).

Interestingly, the spatial regulation of hnRNP members differs depending on both the brain structure and the cell type. To illustrate this, we examine the transcriptional landscape of hnRNPs throughout murine corticogenesis (Telley et al., 2019; Figure 5). The findings revealed that the majority of hnRNP members are expressed throughout the cortical development in various cell populations, including neuronal progenitors (VZ), migrating neurons (IZ), and post-migratory neurons (CP). Strikingly, hnRNPs appear to be more expressed in neuronal progenitors, as evidenced by hnRNPs F, G (RBMX), H2, GRSF1, hnRNP I (PTBP1), L, LL, and M, compared to neurons localized in the CP, except for RALYL from the hnRNP C sub-family (Telley et al., 2019). Again, difference of expression throughout the cortical plate could be noted for different members of the same hnRNP family (Figure 5). This raises the possibility of not completely overlapping function of close paralogues. To further investigate the spatial expression of hnRNPs in different cell types within the brain, we took advantage of the single-cell data resource called CZ CellxGene Discover to compare their expression among cortical progenitors (radial glial cells), cortical neurons (excitatory neurons from different cortical layers, inhibitory interneurons) and glia cells (astrocytes, oligodendrocytes, oligodendrocyte precursors) in human^4^ (Figure 6). The results confirmed the findings from murine corticogenesis, demonstrating that radial glial cells exhibited a higher expression of hnRNPs compared to cerebral cortex neurons. Among the different subtypes of projection neurons (from layer I to layer VI), hnRNPs seem to be similarly expressed except hnRNP DL that is enriched in deep layer neurons (Figure 6). Interestingly, GABAergic interneurons show slightly higher expression of hnRNPs than pyramidal neurons, potentially corroborating the distinct splicing programs identified in glutamatergic and GABAergic neurons (Feng et al., 2021). Specifically, there is higher expression in interneurons for hnRNPs (A0, A1, DL, K, and R), whereas RALYL is more expressed in pyramidal neurons. Neurons from various layers demonstrate robust expression of RALYL. Given the differential expression pattern of RALYL in the different neuronal subtypes, one might consider this hnRNP as a specific marker to transcriptionally differentiate and classify the layer-specific cortical neurons. No significant difference is observed between oligodendrocytes and oligodendrocyte precursors. However, astrocytes clearly exhibit lower expression of all hnRNPs compared to other cell types, thereby corroborating previous observations by Kamma et al., who noted reduced staining in glial cells compare to Purkinje cells (Kamma et al., 1995; Figure 6).

### 3.3 Auto- and cross-regulation

The expression of hnRNP proteins is precisely regulated. hnRNPs expression is occasionally regulated by others splicing factors such as SRp30c (also known as SFRS9) that modulates the alternative splicing of *hnRNP A1* by inhibiting the use of a 3′ splice site (Simard and Chabot, 2002). However, in most cases hnRNP proteins can undergo auto- and cross-regulation, notably through AS-NMD, a mechanism that couples alternative splicing to nonsense-mediated decay to force the production of NMD-sensitive isoforms and thereby adjust the level of protein expression (Ni et al., 2007; Müller-McNicoll et al., 2019). It has been shown that hnRNP A2/B1 alters the splicing of the 3’UTR of its own mRNA, leading to the production of NMD-targeted isoforms (McGlincy et al., 2010). hnRNP I (also known as PTBP1) also binds to its own pre-mRNA to suppress the inclusion of exon 11. This induces a frameshift, resulting in the creation of a premature termination codon in the subsequent exon, consequently directing the mRNA for nonsense-mediated decay (Wollerton et al., 2004). Interestingly, hnRNP I can also regulate the level of its PTBP2 (nPTB) paralogue by promoting the skipping of *PTBP2* (*nPTB*) exon 10 and the subsequent production of an NMD substrate, so that only one of the two paralogues are expressed when both genes are transcribed (Spellman et al., 2007). On the same line, the closely related paralogs hnRNP L and LL (Rossbach et al., 2009), along with hnRNP D (also known as AUF1) and hnRNP DL (Kemmerer et al., 2018) have also been reported to control their own expression as well as that of each other hnRNPs through AS-NMD (Müller-McNicoll et al., 2019). In accordance with cross-regulation among the hnRNP families, analysis of binding sites for various hnRNPs within the genes encoding the different hnRNPs revealed a large network of cross-regulatory interactions between hnRNPs (Huelga et al., 2012). Enlarging the modes of regulation of hnRNPs, it has been shown that TDP-43 binds to its own mRNA through sequences within the CDS and the 3’UTR to target *TDP-43* transcripts to degradation likely via the exosome system (Ayala et al., 2011).

## 4 hnRNPs splicing function in the brain

In this section, we will focus on the historically described function of hnRNPs, the regulation of splicing (Dreyfuss et al., 1993), specifically in the developing brain. Extensive discussion of the other functions of hnRNPs in transcriptional regulation, nucleocytoplasmic transport, mRNA biogenesis (stability, metabolism, localization) and decay, translational regulation, chromatin remodeling or telomere maintenance, can be found in recent reviews (Geuens et al., 2016; Purice and Taylor, 2018; Bampton et al., 2020; Low et al., 2021; Brandão-Teles et al., 2023).

Among hnRNPs, hnRNP I (PTBP1) and PTBP2, members of the hnRNP I family, are widely described as key splicing factors during brain development. One of their main roles is to promote the timely expression of synaptic genes during brain maturation. Indeed, both hnRNP I (PTBP1) and PTBP2 are involved in the regulation of the expression of PSD95, that plays a key role in synapse maturation. In progenitors, they both promote PSD95 mRNA decay by suppressing the splicing of its exon 18. As progenitors differentiate into neurons, both genes are progressively silenced, resulting in exon 18 splicing and subsequent PSD95 expression. In accordance, reintroduction of hnRNP I (PTBP1) or PTBP2 in differentiated neurons inhibits PSD95 expression, impairing glutamatergic synapse development (Zheng et al., 2012). Recent PTBP2 CLIP-seq analysis in both human cortical tissue and neurons derived from induced pluripotent stem cells revealed other synaptic genes as novel PTBP2 targets. This includes *SYNGAP1*, a synaptic gene implicated in a neurodevelopmental disorder (Dawicki-McKenna et al., 2023). It was shown that PTBP2 promotes the inclusion of an alternative 3′ splice site in exon 11 of *SYNGAP1* resulting in the introduction of a premature stop codon and degradation of the *SYNGAP1* mRNA. Of note, hnRNP I (PTBP1) also regulates this splicing event. As for PSD95, progressive downregulation of PTBP proteins as neurons mature drives the increased expression of SYNGAP1 (Dawicki-McKenna et al., 2023). Among the other synapse-associated targets, Dawicki-McKenna et al. also found the glutamate receptor gene *GRIN1* to be spliced by PTBP2. This event involves the inclusion of a previously unannotated alternative exon, resulting in a frameshift in the canonical transcript and reduced expression. Yet, the role of hnRNP I (PTBP1) in the regulation of *GRIN1* has not been addressed (Dawicki-McKenna et al., 2023). Notably, hnRNP I (PTBP1) and PTBP2 could also regulate synapse formation through the regulation of expression of the different neurexins isoforms, the adhesion molecules that shape neuronal synapses (Resnick et al., 2008). Aside from its roles in synapse formation and maturation, PTBP2 plays a role in regulating the timing of axonogenesis, notably by regulating the switch from the long to the short isoforms of Shootin1, that sequentially regulate axon formation and elongation through distinct function on actin cytoskeleton (Zhang et al., 2019). Strikingly, this role is unique to PTBP2. Interestingly, the differences in the splicing regulation patterns of hnRNP I (PTBP1) and PTBP2 have been shown to arise from shift in the expression levels of hnRNP I (PTBP1) and PTBP2 proteins during neuronal differentiation. Indeed, hnRNP I (PTBP1) suppresses the inclusion of alternative exon 10 in the *PTBP2* pre-mRNA, leading to the generation of a premature termination codon and its degradation through NMD (Boutz et al., 2007). Upon differentiation of progenitors to neurons, the repression of hnRNP I (PTBP1) expression is facilitated by the miRNA miR124 (Makeyev et al., 2007) releasing the negative regulation of hnRNP I (PTBP1) on PTBP2 (Boutz et al., 2007). Also, hnRNP I (PTBP1) is critical to maintain the pool of neural progenitor cells (NPCs) by repressing a poison exon in filamin A specifically in NPCs. Not least, a human intronic mutation within a hnRNP I (PTBP1) binding site in the *FLNA* gene that prevents the usual exclusion of the *FLNA* poison exon in NPCs, results in a brain-specific malformation (Zhang et al., 2016). Like hnRNP I (PTBP1), hnRNPs H1/H2 have been shown to regulate the ability of the progenitors to generate neurons. Indeed, hnRNP H1/H2 proteins bind to *TRF2* (telomere repeat-binding factor 2) exon 7, inhibiting its splicing and thereby inhibiting the production of the exon 7 truncated TRF2-S short isoform, that is essential to promote neurogenesis (Grammatikakis et al., 2016). Interestingly, as neurogenesis progresses, there is a gradual decline in the levels of hnRNP H1/H2 proteins, that coincides with an increase in the abundance of TRF2-S. Notably, experimental silencing of hnRNPs H1/H2 leads to elevated levels of TRF2-S, thereby promoting neurogenesis (Grammatikakis et al., 2016).

Other hnRNPs have been identified as important for splicing in a physiological context: (i) *hnRNP U* knockout in mouse dorsal telencephalon leads to numerous alternative splicing events, notably in Doublecortin that controls axon growth and guidance, Siva1 that regulates neural apoptosis and synaptic function, and MDM2, a p53 negative regulator, which is targeted in brain tumor therapy (Sapir et al., 2022); (ii) hnRNP K competes with the constitutive splicing factor U2AF65 to control the splicing of several neuronal genes including *Snap25* (synaptosomal-associated protein 25) during neuronal differentiation (Cao et al., 2012); (iii) whole-genome investigation of alternative splicing has revealed the significant involvement of hnRNP F/H sub-family in the proliferation and differentiation processes of oligodendrocytes (Wang et al., 2012). In addition, hnRNPs F/H play a crucial role in regulating the major proteolipid protein in oligodendrocytes, underscoring its importance in the development and functioning of myelinating cells (Wang et al., 2007).

Splicing function of hnRNPs is also clearly associated to neurodegeneration condition. First, Spinal Muscular Atrophy (SMA), that primarily affects the motor neurons in the spinal cord, involves the splicing function of hnRNPs as key factors. The *SMN2* gene, which encodes the survival motor neuron 2 protein undergoes complex splicing regulation, notably splicing of exon 7. This process involves the intricate interplay of several hnRNP proteins. Among these, hnRNP G (RBMX), hnRNP M, and hnRNP Q, facilitate the inclusion of exon 7 in *SMN2*. Conversely, the depletion of hnRNPs A1/A2 promotes exon 7 inclusion in *SMN2*. Remarkably, *SMN2* is almost identical to *SMN1* gene, which is mutated in SMA. Interestingly, promoting expression of the full SMN2 isoform containing exon 7 in a SMN1 mutated context reduces the severity of SMA (Singh and Singh, 2018; Wirth et al., 2020; Wirth, 2021). Second, as extensively reviewed by Corsi et al. (2022), there are many hnRNP proteins [D, A3, H1, C, R, A2/B1, A1, G (RBMX), E2 (PCBP2), I (PTBP1), and PTBP2] that intricately regulate *MAPT* splicing, impacting the balance between various tau isoforms crucial for normal neuronal function and implicated in neurodegenerative diseases like Alzheimer’s disease. Third, TDP-43 is a central player in the pathogenesis of the neurodegenerative disorder Frontotemporal Dementia-Amyotrophic Lateral Sclerosis (FTD-ALS). Specifically, the mislocalization of TDP-43 in the cytoplasm induces aberrant splicing of several genes: (i) activation of a cryptic splice site in the first intron of *STMN2* gene (encoding Stathmin-2) that compromises axon repair following motor neuron injury in ALS (Klim et al., 2019; Melamed et al., 2019; Baughn et al., 2023), (ii) insertion of a cryptic exon between exon 20 and 21 within the *UNC13A* transcript, a gene that plays important roles in neurotransmitter release at synapses. Consequently, this alternative splicing event generates a premature stop codon and triggers the NMD mechanism to degrade *UNC13A* pre-mRNA (Brown et al., 2022; Ma et al., 2022). Of note, TDP-43 interacts with some hnRNP members (A1, A2/B1, and L) that have been recently shown to also bind *UNC13A* RNA and repress cryptic exon inclusion, independently of TDP-43 (Koike et al., 2023). Accordingly, this has recently been corroborated using a genetically modified neuronal cell line that overexpresses either hnRNP L or a GFP control. They demonstrated that overexpression of hnRNP L decreases the abnormal inclusion of the *UNC13A* cryptic exon in a siRNA *TDP-43* condition and elevates the levels of full-length *UNC13A* in a siRNA scramble condition (Agra Almeida Quadros et al., 2024). They also demonstrated that overexpression of hnRNP L does not correct the splicing defect of the *STMN2* transcripts in a siRNA *TDP-43* condition (Agra Almeida Quadros et al., 2024).

## 5 Functional compensation between hnRNP members

As seen in the previous sections, members of hnRNP sub-families share various structural and functional properties raising the possibility that hnRNPs might have redundant functions. Recent evidence supports a functional compensation between close paralogues. Whether compensatory mechanisms exist across hnRNPs from different sub-families need to be demonstrated.

Mouse genetics have suggested some compensatory mechanisms among hnRNP proteins. First knockin mouse models carrying *HnRnp H2* variants found in patients presenting with neurodevelopmental disorder have been generated, along with *HnRnp H2*-KO mice (Korff et al., 2023). While the knockin mice recapitulated key clinical features observed in human patients, including reduced survival, impaired motor and cognitive functions, the *HnRnp H2*-KO mice displayed no discernible phenotypes. Intriguingly, the KO mice exhibited upregulated expression of hnRNP H1 while knockin mice failed to upregulate hnRNP H1. These findings suggest a compensatory mechanism by hnRNP H1 to counteract the loss of hnRNP H2, implying that the hnRNP H2-related disorder may result from a toxic gain of function or a complex loss of hnRNP H2 function with impaired compensation by hnRNP H1 (Korff et al., 2023). Second, Vuong et al. demonstrated that overexpression of PTBP1 (hnRNP I) rescues the lethality and brain degenerative phenotypes induced by the inactivation of *PTBP2* (*nPTB*) in mice. They further showed that hnRNP I (PTBP1) partly compensates for splicing defect occurring upon *Ptbp2* depletion. More importantly, this compensation occurs when *Ptbp2* is inactivated in dorsal progenitors (Emx1 + cells) but not when *Ptbp2* is inactivated in the whole brain (Nestin + cells), suggesting that the redundancy of the two proteins could be restricted to specific cell types during brain development (Vuong et al., 2016).

Additional work in cellular model confirm a potential redundancy of hnRNPs: (i) in the context of hnRNP G (RBMX) sub-family, the work of David Elliott’s Lab has shown that the defects in splicing induced by the loss of *hnRNP G* (*RBMX*) in HEK293 cells is compensated by the exogenous expression of its 73% identical RBMXL2 retrocopy and even by the more divergent RBMY1A1 protein (Ehrmann et al., 2019; Siachisumo et al., 2023). The fact that two testis-specific proteins can rescue hnRNP G (RBMX) function in a different cellular context, strongly argue for common and conserved function through evolution. Interestingly, several patients carrying mutations in the *hnRNP G* (*RBMX*) gene have been reported to manifest various syndromes, such as Shashi syndrome and Gustavson syndrome (Shashi et al., 2015; Johansson et al., 2023). Yet, there is little phenotypic overlap between the two syndromes, suggesting a distinct disease-causing mechanism. It has been proposed without being demonstrated that phenotypic variations could be linked to hnRNP G (RBMX) retrocopies, particularly RBMXL1 and RBMXL9, which are known to be expressed in the brain (Johansson et al., 2023). As demonstrated for RBMXL2 (Siachisumo et al., 2023), one can hypothesize that those two retrocopies could also compensate for some splicing defects in those two hnRNP G (RBMX) related brain disorders; (ii) in a search for regulator of cell growth, He et al. showed that while knockdown of hnRNP A2 leads to growth defects, hnRNP A1 and hnRNP A3 depletion did not alter growth unless they are simultaneously depleted (He et al., 2005). This result suggests that these two hnRNP proteins that show the highest sequence identity (Ma et al., 2002) may functionally compensate for each other (He et al., 2005); and (iii) hnRNP L (Yu et al., 2009) and its paralog hnRNP LL (Liu et al., 2012) redundantly modulates the splicing of the calcium/calmodulin-dependent protein kinase IV (CaMKIV), a crucial enzyme involved in signal transduction and gene expression regulation. However, it is important to keep in mind that the redundancy could be specific to some mRNAs. Indeed, although the domain architecture between both proteins is highly conserved, with each containing four very similar RRMs, hnRNP L and hnRNP LL are very different in respect of their binding preferences: while hnRNP LL prefers binding to the CANRCA sequence, hnRNP L shows a broader range of preferred target sequences (CANRCA, CAN_2_RCA, and CACA). The biological consequence of the differential sequences preferences of hnRNP L and LL can be evidenced by their distinct binding to the CD45 regulatory element ESS1, that present seven “CA repeat” known to differentially regulate CD45 splicing repression: while both hnRNP L and LL can bind CA6-7 repeat, only hnRNP L binds to CA2-4 repeats (Smith et al., 2013). Another example illustrating the opposite effects of hnRNP L and hnRNP LL is the splicing of the *CHRNA1* gene. While hnRNP L promotes the exclusion of exon P3A in the *CHRNA1* pre-mRNA, hnRNP LL tends to favor its inclusion (Rahman et al., 2013).

In sum, the compensatory mechanisms among hnRNPs seem very complex ranging from broad overlap in their function to compensation of specific splicing event or in specific cellular context. As such, the full characterization of functional compensation between hnRNPs represent a challenge that can be only met by extensive bench work.

## 6 hnRNPs and neurodevelopmental/neurodegenerative disorders

### 6.1 Neurodevelopmental disorders

Growing evidence link variants in multiple *hnRNP* genes to neurodevelopmental disorders (NDD). These disorders encompass a wide spectrum of neurodevelopmental symptoms, including developmental delay, microcephaly, brain anomalies, intellectual disability, and epilepsy (Gillentine et al., 2021), and have been referred as HNRNP-Related Rare Neurodevelopmental Disorders (HNRNP-RNDDs) by the hnRNP family foundation (see text footnote 1) (Gillentine et al., 2021). Though the association with HNRNP-RNDDs have been clearly shown for 8 hnRNPs (detailed below), several other hnRNPs (AB, D, F, H3, UL1, and UL2) are relevant candidate for NDDs (Gillentine et al., 2021), but this needs to be formally demonstrated. Notably, these candidates do not show a similar expression pattern neither in time or space (Figures 4, 5), that would explain their association to disease. Interestingly, although the molecular mechanism underlying HNRNP-RNDDs have not been fully investigated, to date, most of the studies converge toward loss of function effect of the identified variants in hnRNPs. Whether all these disorders are solely caused by the alteration of the canonical splicing function of hnRNPs and how the variants lead to brain phenotype at the cellular level is not known.

#### 6.1.1 hnRNP G

*hnRNP G* is a X-linked gene located at the genetic locus Xq26.3. In line with a key function of hnRNP G (RBMX) in brain development, a hemizygous 23-base pair deletion, resulting in a frameshift mutation and premature termination in the last exon of *hnRNP G* (*RBMX*), has been identified in males from a large family in North Carolina. All affected males present with intellectual disability, craniofacial dysmorphism, and other neurological features. This syndrome was characterized as the Intellectual developmental disorder, X-linked syndromic, Shashi type (Phenotype MIM number: 300986) or HNRNPG-RNDD (Shashi et al., 2000, 2015). At the molecular level the mode of action of these variants has not been identified yet.

Recently, three affected males from a large Swedish family carrying a hemizygous 3-base pair in-frame deletion within exon 5 of the *hnRNP G* gene were diagnosed with Gustavson-type X-linked syndromic intellectual developmental disorder (Gustavson et al., 1993; Johansson et al., 2023). Gustavson syndrome is characterized by microcephaly, severe intellectual disabilities, optic atrophy with visual impairment, hearing loss, spasticity, seizures, and restricted joint mobility and therefore differ from the Shashi syndrome (Shashi et al., 2000, 2015; Johansson et al., 2023). HNRNPG-RNDD spectrum has thus been expanded to Intellectual Developmental Disorder, X-linked syndromic, Gustavson type (Phenotype MIM number: 309555). The 3 bp deletion leads to the removal of the proline at position 162. This in frame deletion of a single aa could impair protein–protein interaction as Pro162 is part of a tri-proline stretch that has been shown to facilitate interaction with SH3 domain-containing proteins. RNA sequencing of SH-SY5Y overexpressing GFP-tagged WT or DelPro162 hnRNP G (RBMX) proteins revealed an enrichment of genes involved in RNA polymerase II transcription among the differentially expressed genes (Gillentine, 2023; Johansson et al., 2023). As mentioned earlier, the most recent retrocopy of hnRNP G, RBMXL1, found on chromosome 1, encodes a protein (Q96E39) with similar expression pattern and high homology with hnRNP G (RBMX) (P38159) (Lingenfelter et al., 2001). Interestingly, although the proline 162 is conserved in RBMXL1, the proline 161 that forms the tri-proline motif in hnRNP G (RBMX) (PPP–160–162) is mutated into a serine (PSP–160–162). It has been suggested that the disruption of the tri-proline motif in the retrocopy excludes the possibility that RBMXL1 compensates for the effects of the hnRNP G (RBMX) variant observed in the patients (Johansson et al., 2023).

#### 6.1.2 hnRNP H2

As *hnRNP G* (*RBMX*), *hnRNP H2* map to the chromosome X (Xq22.1 locus). Unrelated females carrying distinct variants in the *hnRNP H2* gene were identified with developmental delay, intellectual disability, and autism. They were classified under the Intellectual Developmental Disorder, X-linked syndromic, Bain type (Phenotype MIM number: 300986) or HNRNPH2-RNDD (Bain et al., 2016, 2021; Peron et al., 2020). Interestingly, variants affecting conserved residues within the NLS are associated with more severe phenotype compare to variants located outside the NLS (Bain et al., 2016), suggesting that gain of cytoplasmic localization of the protein severely hampers brain development (Bain et al., 2021). Although, no toxic nuclear or cytoplasmic accumulation was observed in muscle tissue biopsies (Bain et al., 2021), investigations of mouse models carrying hnRNP H2 variants, revealed an accumulation of mutant proteins within cytoplasmic RNA granules, confirming a possible gain of function mechanism due to the mislocalization of mutant hnRNP H2 protein (Korff et al., 2023).

While initial studies only reported females patients (Bain et al., 2016, 2021; Peron et al., 2020), some recent studies have documented males carrying pathogenic variants in hnRNP H2 (Harmsen et al., 2019; Jepsen et al., 2019; Somashekar et al., 2020; Kreienkamp et al., 2022). For instance, Harmsen et al. identified a hemizygous *de novo* missense mutation in a young male diagnosed with the Bain type of X-linked syndromic intellectual developmental disorder and presenting with developmental delay, intellectual disability, and progressive microcephaly. This contradicts the initial hypothesis according to which variants in hnRNP H2 are lethal in males during embryonic development (Bain et al., 2016, 2021; Peron et al., 2020).

#### 6.1.3 hnRNP H1

*hnRNP H1* that encodes the close paralogue of hnRNP H2, is found on chromosome 5. A *de novo* heterozygous variant in the PY-NLS (p.R206W) of hnRNP H1 protein has been identified in a young boy diagnosed with a neurodevelopmental disorder characterized by craniofacial dysmorphism and skeletal and ophthalmological defects (Pilch et al., 2018). These latter clinical features being unique to individuals with hnRNP H1 variant, this initial patient (Pilch et al., 2018) as well as 7 other patients carrying the same R206W variant, frameshift variant, in frame deletion or gene duplication (Reichert et al., 2020) were categorized under a related but distinct condition: neurodevelopmental disorder with craniofacial dysmorphism and skeletal defects (Phenotype MIM number: 620083) or HNRNPH1-RNDD. Strikingly, a variant at the corresponding arginine position in hnRNP H2 has also been found to be mutated in individuals with Bain Syndrome (Phenotype MIM number 300986) (Bain et al., 2016), confirming that variants in those two close paralogues lead to distinct NDDs. Of note, the severity of the phenotypes due to pathogenic variants in hnRNP H1 is variable, with variants in the NLS associated with the more severe phenotypes. Mode of action of those variants has not been addressed.

#### 6.1.4 hnRNP C

Recently, hnRNP C has been added to the list of HNRNP-RNDDs and classified under Intellectual developmental disorder, autosomal dominant 74 (Phenotype MIM number: 620688). Two independent studies have identified 13 young individuals, ranging from 17 months to 15 years old (7 males and 6 females), carrying deletions in the C-terminal (5 patients) or N-terminal region (1 patient), frameshift mutations (4 patients), and missense mutations (3 patients) in the *hnRNP C* gene, all variants being found at the heterozygous levels (Kaplanis et al., 2020; Niggl et al., 2023). All patients present with motor and speech delay, intellectual disability, and facial dysmorphisms (Kaplanis et al., 2020; Niggl et al., 2023). Analysis of hnRNP C protein level in iPSCs derived from PBMCs obtained from a patient carrying a C-terminal deletion revealed haploinsufficiency. At the molecular level, *hnRNP C* knockdown in human cell lines or haploinsufficiency in fibroblast cells obtained from a patient with a frameshift mutation lead to defects in the alternative splicing of 60 genes associated with intellectual disability (Niggl et al., 2023). Moreover, *in utero* electroporation (IUE) experiments in mice of two distinct siRNAs against *hnRNP C* gene to deplete *hnRNP C* in cells destined to form the somatosensory cortex at embryonic day E14.5, demonstrated that *hnRNP C*-deficient neurons failed to properly reach the cortical plate compared to the control condition. Further *in vitro* and *in vivo* experiments showed that overexpression of WT hnRNP C phenocopies the loss of hnRNP C function, suggesting that the dose of hnRNP C is critical for proper cortical development (Niggl et al., 2023).

#### 6.1.5 hnRNP U

HNRNPU-related neurodevelopmental disorder (HNRNPU-RNDD) has been extensively studied and documented in numerous publications. Patients were associated to Developmental and epileptic encephalopathy 54 (Phenotype MIM number: 617391) and develop a range of symptoms, typically including moderate to severe intellectual disability, seizures, behavioral abnormalities, speech and language delay as well as craniofacial dysmorphism and agenesis of the corpus callosum (Caliebe et al., 2010; Ballif et al., 2012; Bramswig et al., 2017; Depienne et al., 2017; Leduc et al., 2017; Yates et al., 2017; Durkin et al., 2020; Taylor et al., 2022). A wide range of *de novo* variants have been identified in NDD patients. This includes splice site variants (9), nonsense (14), missense (5), in frame deletion (2), frameshift duplications (3), Frameshift deletion (26) and larger deletion (1), for a total of 57 variants identified to date (Taylor et al., 2022). Those genetics studies strongly suggest that haplinsufficiency is the main mechansism of pathogenecity in HNRNPU variants.

#### 6.1.6 hnRNP R

One study reported four unrelated patients who present with developmental delay, microcephaly, facial dysmorphism and skeletal and brain abnormalities (Phenotype MIM number: 620073) (Duijkers et al., 2019). Authors have identified one missense variant and 2 frameshift variants in the last exon, shown to lead to the production of truncated proteins lacking most of the hnRNP R RGG domain. RNAseq analysis preformed in cells from patients carrying the frameshift variants revealed a strong enrichment of homeobox genes, known for their role in development, among the most deregulated genes. Further candidates-based analysis attributed this HOX deregulation to impaired splicing (Duijkers et al., 2019). To note, a nonsense variant in the last exon has also been identified in a patient presenting with epileptic encephalopathy but also some clinical features overlapping with the 4 other variants (Helbig et al., 2016; Duijkers et al., 2019). The fact that truncated variants leading to very similar proteins lead to different syndrome is puzzling and hamper a clear classification as HNRNPR-related neurodevelopmental disorder (HNRNPR-RNDD).

#### 6.1.7 hnRNP Q

SYNCRIP, also known as hnRNP Q, is also associated with a neurodevelopmental disorder (HNRNPQ-RNDD), characterized by developmental delay, intellectual disability, and autism spectrum disorder accompanied in some cases by malformations of cortical development and myoclonic-atonic epilepsy (Phenotype MIM number: 616686). Eight patients have been identified so far. They all carry *de novo* variants, including frameshift variant (5 patients), missense variant (2), in frame deletion (1) and whole gene deletion (1), suggesting loss of function mechanism (Firth et al., 2009; Rauch et al., 2012; Lelieveld et al., 2016; Guo et al., 2019; Semino et al., 2021). Yet, this has not been tested. To note, SYNCRIP (HNRNP Q) is also part of the proximal 6q loci that have been shown to be deleted in 20 individuals with moderate to severe NDDs (Engwerda et al., 2018).

#### 6.1.8 hnRNP K

Au-Kline syndrome (AKS) or also known as Okamoto syndrome (Phenotype MIM number: 616580), named after the clinicians who first described the pathology (Au et al., 2018; Okamoto, 2019) and characterized by intellectual disability, facial dysmorphisms, and skeletal malformations is caused by mutation in *hnRNP K* and has therefore been added to the HNRNP-RNDD list (Gillentine et al., 2021). The identified *de novo* variants include deletion of a region encompassing hnRNP K (3 individuals), 3 frameshift variants from which two have been experimentally proven to lead to mRNA degradation of the mutant mRNA by NMD and 1 missense mutation, indicating that *hnRNP K* haploinsufficiency is driving neurodevelopmental phenotypes (Lange et al., 2016; Miyake et al., 2017; Au et al., 2018; Okamoto, 2019; Maystadt et al., 2020).

### 6.2 Neurodegenerative disorders

#### 6.2.1 FTLD-ALS spectrum

To date, genetic studies have linked 4 hnRNPs [A1, A2/B1, FUS (hnRNP P2) and TDP-43] to neurodegenerative diseases, including Amyotrophic lateral sclerosis (ALS), Frontotemporal dementia (FTD) and Frontotemporal lobar degeneration (FTLD) that form a clinical disease continuum from motor neuron degenerative disease to dementia (Van Langenhove et al., 2012; Purice and Taylor, 2018). Given that TDP-43 and FUS (hnRNP P2) are key pathological proteins in FTLD-ALS spectrum, they represent the most extensively studied hnRNP proteins associated with neurodegenerative disorders (Bampton et al., 2020).

Although TDP-43 cytoplasmic and nuclear inclusions have been recognized as hallmarks of both FTD and ALS for a long time, it is now shown that genetic variants in TDP-43 account for 1% of all ALS cases and a small number of FTD cases (Sreedharan et al., 2008; Keating et al., 2022). Most of the identified ALS-FTD mutations are missense variants in the TDP-43 C-terminal low complexity domain (LCD) that is involved in protein–protein interaction and phase separation (Keating et al., 2022). The pathogenic effect of those TDP-43 variants has been associated to loss of the nuclear function of TDP-43 as well as gain-of-function in the cytoplasm where it sequesters mRNAs in inclusions (Halliday et al., 2012; Wood et al., 2021). Notably, TDP-43 does not operate independently to facilitate neurodegeneration. Indeed, multiple mass spectrometry analyses have uncovered a close interaction between TDP-43 and numerous hnRNP members [A0, A1, A2/B1, A3, DL, C, E1 (PCBP1), E2 (PCBP2), G (RBMX), H1, I (PTBP1), K, M, P2 (FUS), Q, R, U, UL1, and UL2] (Freibaum et al., 2010; Romano and Buratti, 2013; García Morato et al., 2023). Among these 19 hnRNPs, 11 have been identified as TDP-43 interactors in at least two independent studies. As this intricate interplay between TDP-43 and other hnRNPs plays a critical role in co-regulating RNA splicing targets (see section 4), and as expression levels of certain hnRNPs vary significantly among individuals with FTLD-TDP and control patients (Mohagheghi et al., 2016), TDP-43-hnRNPs cooperation could be central in ALS-FTD disorder. To note, although not associated to any TDP-43 variants, FTLD-TDP, or frontotemporal lobar degeneration with TDP-43 pathology, that is a subtype of FTD, is characterized by the presence of abnormal accumulations of aggregated cytoplasmic TDP-43 in neurons and glia (Chen-Plotkin et al., 2010).

Mutations in hnRNP P2 (FUS) have been identified in approximately 1% of all ALS cases. In the case of FTD, the genetic and pathological involvement of hnRNP P2 (FUS) is still debated (Josephs et al., 2011; Gami-Patel et al., 2016; Nolan et al., 2016; Ishigaki and Sobue, 2018; Kwok et al., 2020). Mutations associated with ALS are distributed all along the *hnRNP P2* (*FUS*) gene. However, there is a cluster of variants in the C-terminal region encompassing the PY-NLS (495–526 aa), whose pathogenicity has been linked to an abnormal accumulation of hnRNP P2 (FUS) in the cytosol (Khalil et al., 2024). As such, FUS-mediated toxicity and associated neurodegeneration is predominantly associated with gain-of-function mechanisms (Sun et al., 2015; Suzuki and Matsuoka, 2015; Scekic-Zahirovic et al., 2016; Sharma et al., 2016; Devoy et al., 2017; Sama et al., 2017; López-Erauskin et al., 2018; An et al., 2019; Tsai et al., 2020). Like TDP-43, hnRNP P2 (FUS) interacts with many hnRNPs [A1, A2/B1, A3, C, D, G (RBMX), H1, H2, K, M, R, U, UL1, hnRNP P2 (FUS) itself] (Kamelgarn et al., 2016; Reber et al., 2016). hnRNPs represent a quarter of the high-confidence hnRNP P2 (FUS) interactors, suggesting a potential collaboration between hnRNPs and hnRNP P2 (FUS) to bind mRNA (Reber et al., 2016). Accordingly, several hnRNPs, like hnRNP A1, C, D, and G (RBMX) were identified in some but not all hnRNP P2 (FUS) pathological deposits in specific brain regions like entorhinal cortex region or hippocampus in postmortem FTD brain (Gami-Patel et al., 2016).

Mutations occurring within the LCD of hnRNP A1 and hnRNP A2/B1 have been linked to both familial and sporadic cases of ALS. However, they represent a very small subset (less than 1%) of both familial and sporadic ALS cases (Bampton et al., 2020; Khalil et al., 2024). Wild-type hnRNP A2/B1 and hnRNP A1 proteins tend to form self-seeding fibrils, a tendency worsened by disease mutations. The identified missense mutations speed up fibril formation and leading to excessive incorporation of hnRNP A2 and hnRNP A1 into stress granules. They also induce the formation of cytoplasmic inclusions in animal models, mimicking human pathology (Kim et al., 2013). However, it is worth noting that mutations in this region are more frequently associated with the pleiotropic degenerative disorder known as multisystem proteinopathy (Kim et al., 2013; Le Ber et al., 2014; Suzuki et al., 2023). Patients with multiple sclerosis (MS) commonly exhibit genomic single nucleotide variants (SNVs) within the nucleocytoplasmic transport M9 domain of the *hnRNP A1* gene, indicating that disrupted hnRNP A1-mediated nucleocytoplasmic transport may contribute to MS pathology (Lee and Levin, 2014). In samples from MS patients, immunofluorescence analysis demonstrates a significant colocalization of hnRNP A1 and TDP-43 within the cytoplasm of neurons in the brain, contrasting with controls (Salapa et al., 2020). Moreover, RNA sequencing in MS brains (Salapa et al., 2024) revealed differential expression of around 550 genes between control and MS samples. 80% of these differentially expressed transcripts had previously shown binding to hnRNP A1. Overall, the findings endorse the notion that issues with RNA regulation stemming from dysfunctional hnRNP A1 play a pivotal role in driving neurodegeneration in MS (Salapa et al., 2024). In addition, hnRNP A1 and hnRNP B1 levels have been shown to be increased in the cerebrospinal fluid of MS patients compared to patients with other neurological disorders (Sueoka et al., 2004). Although hnRNP A3 belongs to the same sub-family as hnRNP A1 and hnRNP A2/B1, it has not yet been linked to multiple sclerosis (Low et al., 2021).

#### 6.2.2 hnRNPs and Alzheimer’s disease

Alzheimer’s disease (AD) is a neurodegenerative disorder characterized by progressive cognitive decline, memory loss, and neuropathological features including the accumulation of amyloid-beta plaques and tangled proteins called Tau fibrils. Multiple lines of evidence have linked hnRNPs to AD: (1) Although there are no reported cases of hnRNP A1-related mutations that lead to AD (Clarke et al., 2021), expression of hnRNP A1 is markedly diminished in the brains of individuals with Alzheimer’s disease (Berson et al., 2012). This could lead to direct impairment of APP and Tau proteins as hnRNP A1 binding sites have been found in introns 6 and 8 of the *APP* pre-mRNA (Donev et al., 2007) and that hnRNP A1 regulates the splicing of Tau (Liu et al., 2020). (2) A proteomic study of 16 human brain tissues from AD patients and age-matched controls revealed a significantly increased expression of hnRNPs C, K, L, M, R, U and UL2, in AD, while the expression level of TDP-43, and hnRNPs AB, A3, DL, and E1 (PCBP1) were decreased (Zhang et al., 2018). (3) Cytoplasmic mis-localization of hnRNP K in neurons of the dentate nucleus was shown in AD postmortem brain samples (Sidhu et al., 2022). Of note, similar hnRNP K mislocalization has been observed in FTLD brain tissue (Sidhu et al., 2022). (4) hnRNP A/B loss in AD is not due to Aβ or tau but rather to deficits in cholinergic signaling and likely triggers the large changes in alternative splicing observed in AD (Berson et al., 2012). (5) hnRNP C competes with FMRP for mRNA binding sites, leading to the upregulation of APP synthesis (Lee et al., 2010). (6) Computational analysis shows hnRNP Q lncRNAs crucial in protein folding and AD association (Ashraf et al., 2019).

## 7 Discussion

Despite significant progress in identifying and classifying hnRNP members, defining their functions in the context of the brain remains challenging due to their multifunctional nature. While splicing regulation is the most well-described function, others remain poorly understood, particularly their roles in the cytoplasm under physiological or pathological conditions such as in ALS and FTD. Recent evidence has highlighted their significance in neurodevelopmental disorders, although they have been less extensively investigated compared to their role in cancer (Figure 1), where hnRNPs serve as promising biomarkers (Zhou et al., 2021; Li et al., 2022; Lu et al., 2022; Mo et al., 2022; Tuersun et al., 2023). Indeed, there is a 41-year gap between the initial discovery of hnRNP proteins (Beyer et al., 1977) and the initiation of the first clinical trial in 2018 (Natural History Study of hnRNP-related Disorders; ClinicalTrials.gov ID: NCT03492060), involving individuals with hnRNP genetic variants and associated neurological comorbidities (Figure 1). Concomitantly to those first clinical trials, the establishment of two HNRNP family foundations, one in the USA (see text footnote 1) and one in Japan (see text footnote 2), gave a significant boost to the hnRNPs research and promises major breakthroughs, as it is in the field of cancer.

hnRNP members not only regulate their own expression but also that of other hnRNP proteins, whether closely related or not, revealing the complexity of interactions within this RNA-binding protein family. Increasing evidence suggests that hnRNPs can compensate for certain functions of closely related members. This observation extends to another organ with high cellular and molecular similarities to the brain: the testis (Matos et al., 2021). For instance, RBMXL2 compensates for the absence of hnRNP G (RBMX) in somatic cells (Siachisumo et al., 2023). This finding aligns with a recent model proposing that RBMXL2 takes over hnRNP G (RBMX) function during meiosis due to the transcriptional inactivation of the X chromosome (Ehrmann et al., 2019). The intricate interplay network among hnRNP proteins not only complicates our understanding of the mechanisms underlying neurological disorders, but also meets a challenge for the development of targeted therapy. On the other hand, the functional redundancy among hnRNP proteins also instills hope for potential treatments using ASO therapeutic strategy, as recently commented by Kelvington and Abel (2023). Finally, it is noteworthy mentioning that the use of hnRNP as new tool for therapeutic strategies is starting to emerge. Indeed, novel CRISPR-Cas9 applications aim to induce specific RNA splicing by fusing a RNA-targeted CAS9 (dCasRx) to hnRNPs, such as hnRNP A1 (Konermann et al., 2018). This was used in patient-derived iPSCs to modify alternative splicing in the *MAPT* gene, aiming to counteract pathogenic mutations associated with Frontotemporal dementia and parkinsonism linked to chromosome 17 (FTDP-17). First results in human cortical neurons show that this strategy successfully restored the balance between the two major Tau isoforms, Tau-4R and Tau-3R (Konermann et al., 2018). This highlights the need for a sound understanding of the physiological function of hnRNPs and the mechanisms related to the alteration of their normal function in pathological conditions.

## Author contributions

PT: Writing – original draft. SF: Writing – review & editing. JG: Writing – review & editing, Supervision.

## Footnotes

^1^https://www.hnrnp.org

^2^https://hnrnpjapan.org

^3^https://apps.kaessmannlab.org/evodevoapp/

^4^https://cellxgene.cziscience.com/gene-expression

## References

[ref1] Abdul-Manan N. O'Malley S. M. Williams K. R. (1996). Origins of binding specificity of the A1 heterogeneous nuclear ribonucleoprotein. Biochemistry 35, 3545–3554. doi: 10.1021/bi952298p, PMID: 8639505

[ref2] Adamson B. Smogorzewska A. Sigoillot F. D. King R. W. Elledge S. J. (2012). A genome-wide homologous recombination screen identifies the RNA-binding protein RBMX as a component of the DNA-damage response. Nat. Cell Biol. 14, 318–328. doi: 10.1038/ncb2426, PMID: 22344029 PMC3290715

[ref3] Aebersold R. Agar J. N. Amster I. J. Baker M. S. Bertozzi C. R. Boja E. S.. (2018). How many human proteoforms are there? Nat. Chem. Biol. 14, 206–214. doi: 10.1038/nchembio.2576, PMID: 29443976 PMC5837046

[ref4] Agra Almeida Quadros A. R. Li Z. Wang X. Ndayambaje I. S. Aryal S. Ramesh N.. (2024). Cryptic splicing of stathmin-2 and UNC13A mRNAs is a pathological hallmark of TDP-43-associated Alzheimer's disease. Acta Neuropathol. 147:9. doi: 10.1007/s00401-023-02655-0, PMID: 38175301 PMC10766724

[ref5] Alfano L. Caporaso A. Altieri A. Dell'aquila M. Landi C. Bini L.. (2019). Depletion of the RNA binding protein HNRNPD impairs homologous recombination by inhibiting DNA-end resection and inducing R-loop accumulation. Nucleic Acids Res. 47, 4068–4085. doi: 10.1093/nar/gkz076, PMID: 30799487 PMC6486545

[ref6] An H. Skelt L. Notaro A. Highley J. R. Fox A. H. La Bella V.. (2019). ALS-linked FUS mutations confer loss and gain of function in the nucleus by promoting excessive formation of dysfunctional paraspeckles. Acta Neuropathol. Commun. 7:7. doi: 10.1186/s40478-019-0658-x, PMID: 30642400 PMC6330737

[ref7] Andersen J. S. Lyon C. E. Fox A. H. Leung A. K. Lam Y. W. Steen H.. (2002). Directed proteomic analysis of the human nucleolus. Curr. Biol. 12, 1–11. doi: 10.1016/S0960-9822(01)00650-9, PMID: 11790298

[ref8] Asadi M. R. Sadat Moslehian M. Sabaie H. Jalaiei A. Ghafouri-Fard S. Taheri M.. (2021). Stress granules and neurodegenerative disorders: a scoping review. Front. Aging Neurosci. 13:650740. doi: 10.3389/fnagi.2021.650740, PMID: 34248597 PMC8261063

[ref9] Ashraf G. M. Ganash M. Athanasios A. (2019). Computational analysis of non-coding RNAs in Alzheimer's disease. Bioinformation 15, 351–357. doi: 10.6026/97320630015351, PMID: 31249438 PMC6589468

[ref10] Au P. Y. B. Goedhart C. Ferguson M. Breckpot J. Devriendt K. Wierenga K.. (2018). Phenotypic spectrum of Au-Kline syndrome: a report of six new cases and review of the literature. Eur. J. Hum. Genet. 26, 1272–1281. doi: 10.1038/s41431-018-0187-2, PMID: 29904177 PMC6117294

[ref11] Aviner R. Hofmann S. Elman T. Shenoy A. Geiger T. Elkon R.. (2017). Proteomic analysis of polyribosomes identifies splicing factors as potential regulators of translation during mitosis. Nucleic Acids Res. 45, 5945–5957. doi: 10.1093/nar/gkx326, PMID: 28460002 PMC5449605

[ref12] Ayala Y. M. De Conti L. Avendaño-Vázquez S. E. Dhir A. Romano M. D'ambrogio A.. (2011). TDP-43 regulates its mRNA levels through a negative feedback loop. EMBO J. 30, 277–288. doi: 10.1038/emboj.2010.310, PMID: 21131904 PMC3025456

[ref13] Bain J. M. Cho M. T. Telegrafi A. Wilson A. Brooks S. Botti C.. (2016). Variants in HNRNPH2 on the X chromosome are associated with a neurodevelopmental disorder in females. Am. J. Hum. Genet. 99, 728–734. doi: 10.1016/j.ajhg.2016.06.028, PMID: 27545675 PMC5011042

[ref14] Bain J. M. Thornburg O. Pan C. Rome-Martin D. Boyle L. Fan X.. (2021). Detailed clinical and psychological phenotype of the X-linked HNRNPH2-related neurodevelopmental disorder. Neurol Genet 7:e551. doi: 10.1212/NXG.0000000000000551, PMID: 33728377 PMC7954461

[ref15] Ballif B. C. Rosenfeld J. A. Traylor R. Theisen A. Bader P. I. Ladda R. L.. (2012). High-resolution array CGH defines critical regions and candidate genes for microcephaly, abnormalities of the corpus callosum, and seizure phenotypes in patients with microdeletions of 1q43q44. Hum. Genet. 131, 145–156. doi: 10.1007/s00439-011-1073-y, PMID: 21800092

[ref16] Bampton A. Gittings L. M. Fratta P. Lashley T. Gatt A. (2020). The role of hnRNPs in frontotemporal dementia and amyotrophic lateral sclerosis. Acta Neuropathol. 140, 599–623. doi: 10.1007/s00401-020-02203-0 32748079 PMC7547044

[ref17] Baughn M. W. Melamed Z. López-Erauskin J. Beccari M. S. Ling K. Zuberi A.. (2023). Mechanism of STMN2 cryptic splice-polyadenylation and its correction for TDP-43 proteinopathies. Science 379, 1140–1149. doi: 10.1126/science.abq5622, PMID: 36927019 PMC10148063

[ref18] Berson A. Barbash S. Shaltiel G. Goll Y. Hanin G. Greenberg D. S.. (2012). Cholinergic-associated loss of hnRNP-A/B in Alzheimer's disease impairs cortical splicing and cognitive function in mice. EMBO Mol. Med. 4, 730–742. doi: 10.1002/emmm.201100995, PMID: 22628224 PMC3494073

[ref19] Beyer A. L. Christensen M. E. Walker B. W. Lestourgeon W. M. (1977). Identification and characterization of the packaging proteins of core 40S hnRNP particles. Cell 11, 127–138. doi: 10.1016/0092-8674(77)90323-3, PMID: 872217

[ref20] Biamonti G. Riva S. (1994). New insights into the auxiliary domains of eukaryotic RNA binding proteins. FEBS Lett. 340, 1–8. doi: 10.1016/0014-5793(94)80162-2, PMID: 7509757

[ref21] Biamonti G. Ruggiu M. Saccone S. Della Valle G. Riva S. (1994). Two homologous genes, originated by duplication, encode the human hnRNP proteins A2 and A1. Nucleic Acids Res. 22, 1996–2002. doi: 10.1093/nar/22.11.1996, PMID: 8029005 PMC308112

[ref22] Bolinger C. Boris-Lawrie K. (2009). Mechanisms employed by retroviruses to exploit host factors for translational control of a complicated proteome. Retrovirology 6:8. doi: 10.1186/1742-4690-6-8, PMID: 19166625 PMC2657110

[ref23] Boudreault S. Roy P. Lemay G. Bisaillon M. (2019). Viral modulation of cellular RNA alternative splicing: A new key player in virus-host interactions? Wiley Interdiscip Rev RNA 10:e1543. doi: 10.1002/wrna.1543, PMID: 31034770 PMC6767064

[ref24] Bourgeois B. Hutten S. Gottschalk B. Hofweber M. Richter G. Sternat J.. (2020). Nonclassical nuclear localization signals mediate nuclear import of CIRBP. Proc. Natl. Acad. Sci. USA 117, 8503–8514. doi: 10.1073/pnas.1918944117, PMID: 32234784 PMC7165476

[ref25] Boutz P. L. Stoilov P. Li Q. Lin C. H. Chawla G. Ostrow K.. (2007). A post-transcriptional regulatory switch in polypyrimidine tract-binding proteins reprograms alternative splicing in developing neurons. Genes Dev. 21, 1636–1652. doi: 10.1101/gad.1558107, PMID: 17606642 PMC1899473

[ref26] Bramswig N. C. Lüdecke H. J. Hamdan F. F. Altmüller J. Beleggia F. Elcioglu N. H.. (2017). Heterozygous HNRNPU variants cause early onset epilepsy and severe intellectual disability. Hum. Genet. 136, 821–834. doi: 10.1007/s00439-017-1795-6, PMID: 28393272

[ref27] Brandão-Teles C. Antunes A. De Moraes Vrechi T. A. Martins-De-Souza D. (2023). The roles of hnRNP family in the brain and brain-related disorders. Mol. Neurobiol. 61, 3578–3595. doi: 10.1007/s12035-023-03747-4 37999871

[ref28] Briese M. Saal-Bauernschubert L. Ji C. Moradi M. Ghanawi H. Uhl M.. (2018). hnRNP R and its main interactor, the noncoding RNA 7SK, coregulate the axonal transcriptome of motoneurons. Proc. Natl. Acad. Sci. USA 115, E2859–e2868. doi: 10.1073/pnas.1721670115, PMID: 29507242 PMC5866599

[ref29] Britton S. Dernoncourt E. Delteil C. Froment C. Schiltz O. Salles B.. (2014). DNA damage triggers SAF-A and RNA biogenesis factors exclusion from chromatin coupled to R-loops removal. Nucleic Acids Res. 42, 9047–9062. doi: 10.1093/nar/gku601, PMID: 25030905 PMC4132723

[ref30] Brown A. L. Wilkins O. G. Keuss M. J. Hill S. E. Zanovello M. Lee W. C.. (2022). TDP-43 loss and ALS-risk SNPs drive mis-splicing and depletion of UNC13A. Nature 603, 131–137. doi: 10.1038/s41586-022-04436-3, PMID: 35197628 PMC8891020

[ref31] Brunetti J. E. Scolaro L. A. Castilla V. (2015). The heterogeneous nuclear ribonucleoprotein K (hnRNP K) is a host factor required for dengue virus and Junín virus multiplication. Virus Res. 203, 84–91. doi: 10.1016/j.virusres.2015.04.001, PMID: 25865411

[ref32] Burnham A. J. Gong L. Hardy R. W. (2007). Heterogeneous nuclear ribonuclear protein K interacts with Sindbis virus nonstructural proteins and viral subgenomic mRNA. Virology 367, 212–221. doi: 10.1016/j.virol.2007.05.008, PMID: 17561226

[ref33] Busch A. Hertel K. J. (2012). Evolution of SR protein and hnRNP splicing regulatory factors. Wiley Interdiscip Rev RNA 3, 1–12. doi: 10.1002/wrna.100, PMID: 21898828 PMC3235224

[ref34] Calabretta S. Richard S. (2015). Emerging roles of disordered sequences in RNA-binding proteins. Trends Biochem. Sci. 40, 662–672. doi: 10.1016/j.tibs.2015.08.012 26481498

[ref35] Caliebe A. Kroes H. Y. van der Smagt J. J. Martin-Subero J. I. Tönnies H. van ‘t Slot R.. (2010). Four patients with speech delay, seizures and variable corpus callosum thickness sharing a 0.440 Mb deletion in region 1q44 containing the HNRPU gene. Eur. J. Med. Genet. 53, 179–185. doi: 10.1016/j.ejmg.2010.04.001, PMID: 20382278

[ref36] Cao W. Razanau A. Feng D. Lobo V. G. Xie J. (2012). Control of alternative splicing by forskolin through hnRNP K during neuronal differentiation. Nucleic Acids Res. 40, 8059–8071. doi: 10.1093/nar/gks504, PMID: 22684629 PMC3439897

[ref37] Cappelli S. Romano M. Buratti E. (2018). Systematic analysis of gene expression profiles controlled by hnRNP Q and hnRNP R, two closely related human RNA binding proteins implicated in mRNA processing mechanisms. Front. Mol. Biosci. 5:79. doi: 10.3389/fmolb.2018.00079, PMID: 30214903 PMC6125337

[ref38] Cardoso-Moreira M. Halbert J. Valloton D. Velten B. Chen C. Shao Y.. (2019). Gene expression across mammalian organ development. Nature 571, 505–509. doi: 10.1038/s41586-019-1338-5, PMID: 31243369 PMC6658352

[ref39] Cartegni L. Maconi M. Morandi E. Cobianchi F. Riva S. Biamonti G. (1996). hnRNP A1 selectively interacts through its Gly-rich domain with different RNA-binding proteins. J. Mol. Biol. 259, 337–348. doi: 10.1006/jmbi.1996.0324, PMID: 8676373

[ref40] Chaudhury A. Chander P. Howe P. H. (2010). Heterogeneous nuclear ribonucleoproteins (hnRNPs) in cellular processes: focus on hnRNP E1's multifunctional regulatory roles. RNA 16, 1449–1462. doi: 10.1261/rna.2254110, PMID: 20584894 PMC2905745

[ref41] Chen T. M. Lai M. C. Li Y. H. Chan Y. L. Wu C. H. Wang Y. M.. (2019). hnRNPM induces translation switch under hypoxia to promote colon cancer development. EBioMedicine 41, 299–309. doi: 10.1016/j.ebiom.2019.02.059, PMID: 30852162 PMC6444133

[ref42] Chen-Plotkin A. S. Lee V. M. Trojanowski J. Q. (2010). TAR DNA-binding protein 43 in neurodegenerative disease. Nat. Rev. Neurol. 6, 211–220. doi: 10.1038/nrneurol.2010.18, PMID: 20234357 PMC2892118

[ref43] Cheunim T. Zhang J. Milligan S. G. Mcphillips M. G. Graham S. V. (2008). The alternative splicing factor hnRNP A1 is up-regulated during virus-infected epithelial cell differentiation and binds the human papillomavirus type 16 late regulatory element. Virus Res. 131, 189–198. doi: 10.1016/j.virusres.2007.09.006, PMID: 17950949 PMC2635527

[ref44] Cho S. J. Jung Y. S. Chen X. (2013). Poly (C)-binding protein 1 regulates p63 expression through mRNA stability. PLoS One 8:e71724. doi: 10.1371/journal.pone.0071724, PMID: 23940783 PMC3737132

[ref45] Choi Y. D. Dreyfuss G. (1984). Isolation of the heterogeneous nuclear RNA-ribonucleoprotein complex (hnRNP): a unique supramolecular assembly. Proc. Natl. Acad. Sci. USA 81, 7471–7475. doi: 10.1073/pnas.81.23.7471 6594697 PMC392168

[ref46] Cichocka M. Karlik A. Plewka P. Gawade K. Stępień A. Świergiel P.. (2022). The novel role of hnRNP UL1 in human cell nucleoli. Int. J. Biol. Sci. 18, 4809–4823. doi: 10.7150/ijbs.75084, PMID: 35982897 PMC9379416

[ref47] Clarke J. P. Thibault P. A. Salapa H. E. Levin M. C. (2021). A comprehensive analysis of the role of hnRNP A1 function and dysfunction in the pathogenesis of neurodegenerative disease. Front. Mol. Biosci. 8:659610. doi: 10.3389/fmolb.2021.659610, PMID: 33912591 PMC8072284

[ref48] Corre M. Boehm V. Besic V. Kurowska A. Viry A. Mohammad A.. (2023). Alternative splicing induced by bacterial pore-forming toxins sharpens CIRBP-mediated cell response to Listeria infection. Nucleic Acids Res. 51, 12459–12475. doi: 10.1093/nar/gkad1033, PMID: 37941135 PMC10711537

[ref9001] Corsi A. Bombieri C. Valenti M. T. Romanelli M. G. (2022). Tau Isoforms: Gaining Insight into MAPT Alternative Splicing. International Journal Of Molecular Sciences 23, 15383. doi: 10.3390/ijms232315383, PMID: 36499709 PMC9735940

[ref49] D'ambrogio A. Buratti E. Stuani C. Guarnaccia C. Romano M. Ayala Y. M.. (2009). Functional mapping of the interaction between TDP-43 and hnRNP A2 *in vivo*. Nucleic Acids Res. 37, 4116–4126. doi: 10.1093/nar/gkp342, PMID: 19429692 PMC2709582

[ref50] Dawicki-Mckenna J. M. Felix A. J. Waxman E. A. Cheng C. Amado D. A. Ranum P. T.. (2023). Mapping PTBP2 binding in human brain identifies SYNGAP1 as a target for therapeutic splice switching. Nat. Commun. 14:2628. doi: 10.1038/s41467-023-38273-3, PMID: 37149717 PMC10164156

[ref51] Decorsière A. Cayrel A. Vagner S. Millevoi S. (2011). Essential role for the interaction between hnRNP H/F and a G quadruplex in maintaining p53 pre-mRNA 3′-end processing and function during DNA damage. Genes Dev. 25, 220–225. doi: 10.1101/gad.607011, PMID: 21289067 PMC3034896

[ref52] Depienne C. Nava C. Keren B. Heide S. Rastetter A. Passemard S.. (2017). Genetic and phenotypic dissection of 1q43q44 microdeletion syndrome and neurodevelopmental phenotypes associated with mutations in ZBTB18 and HNRNPU. Hum. Genet. 136, 463–479. doi: 10.1007/s00439-017-1772-0, PMID: 28283832 PMC5360844

[ref53] Dettori L. G. Torrejon D. Chakraborty A. Dutta A. Mohamed M. Papp C.. (2021). A tale of loops and tails: the role of intrinsically disordered protein regions in R-loop recognition and phase separation. Front. Mol. Biosci. 8:691694. doi: 10.3389/fmolb.2021.691694 34179096 PMC8222781

[ref54] Devoy A. Kalmar B. Stewart M. Park H. Burke B. Noy S. J.. (2017). Humanized mutant FUS drives progressive motor neuron degeneration without aggregation in 'FUSDelta14' knockin mice. Brain 140, 2797–2805. doi: 10.1093/brain/awx248, PMID: 29053787 PMC5841203

[ref55] Dhillon P. Tandra V. N. Chorghade S. G. Namsa N. D. Sahoo L. Rao C. D. (2018). Cytoplasmic relocalization and colocalization with viroplasms of host cell proteins, and their role in rotavirus infection. J. Virol. 92:e00612-18. doi: 10.1128/JVI.00612-18, PMID: 29769336 PMC6052293

[ref56] Ding J. Hayashi M. K. Zhang Y. Manche L. Krainer A. R. Xu R. M. (1999). Crystal structure of the two-RRM domain of hnRNP A1 (UP1) complexed with single-stranded telomeric DNA. Genes Dev. 13, 1102–1115. doi: 10.1101/gad.13.9.1102, PMID: 10323862 PMC316951

[ref57] Donev R. Newall A. Thome J. Sheer D. (2007). A role for SC35 and hnRNPA1 in the determination of amyloid precursor protein isoforms. Mol. Psychiatry 12, 681–690. doi: 10.1038/sj.mp.4001971, PMID: 17353911 PMC2684093

[ref58] Dowling D. Nasr-Esfahani S. Tan C. H. O'Brien K. Howard J. L. Jans D. A.. (2008). HIV-1 infection induces changes in expression of cellular splicing factors that regulate alternative viral splicing and virus production in macrophages. Retrovirology 5:18. doi: 10.1186/1742-4690-5-18, PMID: 18241354 PMC2267807

[ref59] Dreyfuss G. Kim V. N. Kataoka N. (2002). Messenger-RNA-binding proteins and the messages they carry. Nat. Rev. Mol. Cell Biol. 3, 195–205. doi: 10.1038/nrm760, PMID: 11994740

[ref60] Dreyfuss G. Matunis M. J. Piñol-Roma S. Burd C. G. (1993). hnRNP proteins and the biogenesis of mRNA. Annu. Rev. Biochem. 62, 289–321. doi: 10.1146/annurev.bi.62.070193.001445 8352591

[ref61] Dudman J. Qi X. (2020). Stress granule dysregulation in amyotrophic lateral sclerosis. Front. Cell. Neurosci. 14:598517. doi: 10.3389/fncel.2020.598517, PMID: 33281563 PMC7705167

[ref62] Duijkers F. A. Mcdonald A. Janssens G. E. Lezzerini M. Jongejan A. Van Koningsbruggen S.. (2019). HNRNPR variants that impair Homeobox gene expression drive developmental disorders in humans. Am. J. Hum. Genet. 104, 1040–1059. doi: 10.1016/j.ajhg.2019.03.024, PMID: 31079900 PMC6556882

[ref63] Durkin A. Albaba S. Fry A. E. Morton J. E. Douglas A. Beleza A.. (2020). Clinical findings of 21 previously unreported probands with HNRNPU-related syndrome and comprehensive literature review. Am. J. Med. Genet. A 182, 1637–1654. doi: 10.1002/ajmg.a.61599, PMID: 32319732

[ref64] Ehrmann I. Crichton J. H. Gazzara M. R. James K. Liu Y. Grellscheid S. N.. (2019). An ancient germ cell-specific RNA-binding protein protects the germline from cryptic splice site poisoning. eLife 8:e39304. doi: 10.7554/eLife.39304, PMID: 30674417 PMC6345566

[ref65] Ehrmann I. Dalgliesh C. Tsaousi A. Paronetto M. P. Heinrich B. Kist R.. (2008). Haploinsufficiency of the germ cell-specific nuclear RNA binding protein hnRNP G-T prevents functional spermatogenesis in the mouse. Hum. Mol. Genet. 17, 2803–2818. doi: 10.1093/hmg/ddn179 18562473

[ref66] Elliott D. J. (2004). The role of potential splicing factors including RBMY, RBMX, hnRNPG-T and STAR proteins in spermatogenesis. Int. J. Androl. 27, 328–334. doi: 10.1111/j.1365-2605.2004.00496.x, PMID: 15595951

[ref67] Elliott D. J. Dalgliesh C. Hysenaj G. Ehrmann I. (2019). RBMX family proteins connect the fields of nuclear RNA processing, disease and sex chromosome biology. Int. J. Biochem. Cell Biol. 108, 1–6. doi: 10.1016/j.biocel.2018.12.014, PMID: 30593955

[ref68] Elliott D. J. Oghene K. Makarov G. Makarova O. Hargreave T. B. Chandley A. C.. (1998). Dynamic changes in the subnuclear organisation of pre-mRNA splicing proteins and RBM during human germ cell development. J. Cell Sci. 111, 1255–1265. doi: 10.1242/jcs.111.9.1255, PMID: 9547301

[ref69] Elliott D. J. Venables J. P. Newton C. S. Lawson D. Boyle S. Eperon I. C.. (2000). An evolutionarily conserved germ cell-specific hnRNP is encoded by a retrotransposed gene. Hum. Mol. Genet. 9, 2117–2124. doi: 10.1093/hmg/9.14.2117, PMID: 10958650

[ref70] Engeland C. E. Brown N. P. Börner K. Schümann M. Krause E. Kaderali L.. (2014). Proteome analysis of the HIV-1 gag interactome. Virology 460-461, 194–206. doi: 10.1016/j.virol.2014.04.038, PMID: 25010285

[ref71] Engwerda A. Frentz B. Den Ouden A. L. Flapper B. C. T. Swertz M. A. Gerkes E. H.. (2018). The phenotypic spectrum of proximal 6q deletions based on a large cohort derived from social media and literature reports. Eur. J. Hum. Genet. 26, 1478–1489. doi: 10.1038/s41431-018-0172-9, PMID: 29904178 PMC6138703

[ref72] Evans J. R. Mitchell S. A. Spriggs K. A. Ostrowski J. Bomsztyk K. Ostarek D.. (2003). Members of the poly (rC) binding protein family stimulate the activity of the c-myc internal ribosome entry segment in vitro and in vivo. Oncogene 22, 8012–8020. doi: 10.1038/sj.onc.1206645, PMID: 12970749

[ref73] Ezkurdia I. Del Pozo A. Frankish A. Rodriguez J. M. Harrow J. Ashman K.. (2012). Comparative proteomics reveals a significant bias toward alternative protein isoforms with conserved structure and function. Mol. Biol. Evol. 29, 2265–2283. doi: 10.1093/molbev/mss100, PMID: 22446687 PMC3424414

[ref74] Feng X. Li H. (2021). Higher rates of processed pseudogene Acquisition in Humans and Three Great Apes Revealed by Long-read assemblies. Mol. Biol. Evol. 38, 2958–2966. doi: 10.1093/molbev/msab062, PMID: 33681998 PMC8661421

[ref75] Feng H. Moakley D. F. Chen S. Mckenzie M. G. Menon V. Zhang C. (2021). Complexity and graded regulation of neuronal cell-type-specific alternative splicing revealed by single-cell RNA sequencing. Proc. Natl. Acad. Sci. USA 118:e2013056118. doi: 10.1073/pnas.2013056118 33674385 PMC7958184

[ref76] Fialcowitz E. J. Brewer B. Y. Keenan B. P. Wilson G. M. (2005). A hairpin-like structure within an AU-rich mRNA-destabilizing element regulates trans-factor binding selectivity and mRNA decay kinetics. J. Biol. Chem. 280, 22406–22417. doi: 10.1074/jbc.M500618200, PMID: 15809297 PMC1553220

[ref77] Firth H. V. Richards S. M. Bevan A. P. Clayton S. Corpas M. Rajan D.. (2009). DECIPHER: database of chromosomal imbalance and phenotype in humans using Ensembl resources. Am. J. Hum. Genet. 84, 524–533. doi: 10.1016/j.ajhg.2009.03.010, PMID: 19344873 PMC2667985

[ref78] Freibaum B. D. Chitta R. K. High A. A. Taylor J. P. (2010). Global analysis of TDP-43 interacting proteins reveals strong association with RNA splicing and translation machinery. J. Proteome Res. 9, 1104–1120. doi: 10.1021/pr901076y, PMID: 20020773 PMC2897173

[ref79] Friend L. R. Han S. P. Rothnagel J. A. Smith R. (2008). Differential subnuclear localisation of hnRNPs A/B is dependent on transcription and cell cycle stage. Biochim. Biophys. Acta 1783, 1972–1980. doi: 10.1016/j.bbamcr.2008.05.021, PMID: 18588922

[ref80] Fukuda N. Fukuda T. Percipalle P. Oda K. Takei N. Czaplinski K.. (2023). Axonal mRNA binding of hnRNP A/B is crucial for axon targeting and maturation of olfactory sensory neurons. Cell Rep. 42:112398. doi: 10.1016/j.celrep.2023.112398, PMID: 37083330

[ref81] Fukuda T. Naiki T. Saito M. Irie K. (2009). hnRNP K interacts with RNA binding motif protein 42 and functions in the maintenance of cellular ATP level during stress conditions. Genes Cells 14, 113–128. doi: 10.1111/j.1365-2443.2008.01256.x, PMID: 19170760

[ref82] Gagné M. Deshaies J. E. Sidibé H. Benchaar Y. Arbour D. Dubinski A.. (2021). hnRNP A1B, a splice variant of HNRNPA1, is spatially and temporally regulated. Front. Neurosci. 15:724307. doi: 10.3389/fnins.2021.724307, PMID: 34630013 PMC8498194

[ref83] Gamarnik A. V. Andino R. (1997). Two functional complexes formed by KH domain containing proteins with the 5′ noncoding region of poliovirus RNA. RNA 3, 882–892, PMID: 9257647 PMC1369533

[ref84] Gami-Patel P. Bandopadhyay R. Brelstaff J. Revesz T. Lashley T. (2016). The presence of heterogeneous nuclear ribonucleoproteins in frontotemporal lobar degeneration with FUS-positive inclusions. Neurobiol. Aging 46, 192–203. doi: 10.1016/j.neurobiolaging.2016.07.004, PMID: 27500866

[ref85] García Morato J. Gloeckner C. J. Kahle P. J. (2023). Proteomics elucidating physiological and pathological functions of TDP-43. Proteomics 23:e2200410. doi: 10.1002/pmic.202200410 37671599

[ref86] Ge Z. Quek B. L. Beemon K. L. Hogg J. R. (2016). Polypyrimidine tract binding protein 1 protects mRNAs from recognition by the nonsense-mediated mRNA decay pathway. eLife 5:e11155. doi: 10.7554/eLife.11155, PMID: 26744779 PMC4764554

[ref87] Geissler R. Simkin A. Floss D. Patel R. Fogarty E. A. Scheller J.. (2016). A widespread sequence-specific mRNA decay pathway mediated by hnRNPs A1 and A2/B1. Genes Dev. 30, 1070–1085. doi: 10.1101/gad.277392.116, PMID: 27151978 PMC4863738

[ref88] Geuens T. Bouhy D. Timmerman V. (2016). The hnRNP family: insights into their role in health and disease. Hum. Genet. 135, 851–867. doi: 10.1007/s00439-016-1683-5, PMID: 27215579 PMC4947485

[ref89] Ghanawi H. Hennlein L. Zare A. Bader J. Salehi S. Hornburg D.. (2021). Loss of full-length hnRNP R isoform impairs DNA damage response in motoneurons by inhibiting Yb1 recruitment to chromatin. Nucleic Acids Res. 49, 12284–12305. doi: 10.1093/nar/gkab1120, PMID: 34850154 PMC8643683

[ref90] Gillentine M. A. (2023). Comment on Gustavson syndrome is caused by an in-frame deletion in RBMX associated with potentially disturbed SH3 domain interactions. Eur. J. Hum. Genet. 32, 253–256. 38017187 10.1038/s41431-023-01498-3 PMC10923807

[ref91] Gillentine M. A. Wang T. Hoekzema K. Rosenfeld J. Liu P. Guo H.. (2021). Rare deleterious mutations of HNRNP genes result in shared neurodevelopmental disorders. Genome Med. 13:63. doi: 10.1186/s13073-021-00870-6, PMID: 33874999 PMC8056596

[ref92] Glinka M. Herrmann T. Funk N. Havlicek S. Rossoll W. Winkler C.. (2010). The heterogeneous nuclear ribonucleoprotein-R is necessary for axonal beta-actin mRNA translocation in spinal motor neurons. Hum. Mol. Genet. 19, 1951–1966. doi: 10.1093/hmg/ddq073, PMID: 20167579

[ref93] Godet A. C. David F. Hantelys F. Tatin F. Lacazette E. Garmy-Susini B.. (2019). IRES trans-acting factors, key actors of the stress response. Int. J. Mol. Sci. 20:924. doi: 10.3390/ijms20040924, PMID: 30791615 PMC6412753

[ref94] Grabowski P. J. Black D. L. (2001). Alternative RNA splicing in the nervous system. Prog. Neurobiol. 65, 289–308. doi: 10.1016/S0301-0082(01)00007-7 11473790

[ref95] Grammatikakis I. Zhang P. Panda A. C. Kim J. Maudsley S. Abdelmohsen K.. (2016). Alternative splicing of neuronal differentiation factor TRF2 regulated by HNRNPH1/H2. Cell Rep. 15, 926–934. doi: 10.1016/j.celrep.2016.03.080, PMID: 27117401 PMC4856555

[ref96] Gueroussov S. Gonatopoulos-Pournatzis T. Irimia M. Raj B. Lin Z. Y. Gingras A. C.. (2015). An alternative splicing event amplifies evolutionary differences between vertebrates. Science 349, 868–873. doi: 10.1126/science.aaa8381, PMID: 26293963

[ref97] Guil S. Long J. C. Cáceres J. F. (2006). hnRNP A1 relocalization to the stress granules reflects a role in the stress response. Mol. Cell. Biol. 26, 5744–5758. doi: 10.1128/MCB.00224-06, PMID: 16847328 PMC1592774

[ref98] Guo H. Duyzend M. H. Coe B. P. Baker C. Hoekzema K. Gerdts J.. (2019). Genome sequencing identifies multiple deleterious variants in autism patients with more severe phenotypes. Genet. Med. 21, 1611–1620. doi: 10.1038/s41436-018-0380-2, PMID: 30504930 PMC6546556

[ref99] Gustavson K. H. Annerén G. Malmgren H. Dahl N. Ljunggren C. G. Bäckman H. (1993). New X-linked syndrome with severe mental retardation, severely impaired vision, severe hearing defect, epileptic seizures, spasticity, restricted joint mobility, and early death. Am. J. Med. Genet. 45, 654–658. doi: 10.1002/ajmg.1320450527, PMID: 8456840

[ref100] Halliday G. Bigio E. H. Cairns N. J. Neumann M. Mackenzie I. R. Mann D. M. (2012). Mechanisms of disease in frontotemporal lobar degeneration: gain of function versus loss of function effects. Acta Neuropathol. 124, 373–382. doi: 10.1007/s00401-012-1030-4 22878865 PMC3445027

[ref101] Han S. P. Kassahn K. S. Skarshewski A. Ragan M. A. Rothnagel J. A. Smith R. (2010a). Functional implications of the emergence of alternative splicing in hnRNP A/B transcripts. RNA 16, 1760–1768. doi: 10.1261/rna.2142810, PMID: 20651029 PMC2924535

[ref102] Han S. P. Tang Y. H. Smith R. (2010b). Functional diversity of the hnRNPs: past, present and perspectives. Biochem. J. 430, 379–392. doi: 10.1042/BJ20100396, PMID: 20795951

[ref103] Harmsen S. Buchert R. Mayatepek E. Haack T. B. Distelmaier F. (2019). Bain type of X-linked syndromic mental retardation in boys. Clin. Genet. 95, 734–735. doi: 10.1111/cge.13524, PMID: 30887513

[ref104] Hatfield J. T. Rothnagel J. A. Smith R. (2002). Characterization of the mouse hnRNP A2/B1/B0 gene and identification of processed pseudogenes. Gene 295, 33–42. doi: 10.1016/S0378-1119(02)00800-4, PMID: 12242009

[ref105] He Y. Brown M. A. Rothnagel J. A. Saunders N. A. Smith R. (2005). Roles of heterogeneous nuclear ribonucleoproteins A and B in cell proliferation. J. Cell Sci. 118, 3173–3183. doi: 10.1242/jcs.02448 16014382

[ref106] Helbig K. L. Farwell Hagman K. D. Shinde D. N. Mroske C. Powis Z. Li S.. (2016). Diagnostic exome sequencing provides a molecular diagnosis for a significant proportion of patients with epilepsy. Genet. Med. 18, 898–905. doi: 10.1038/gim.2015.186, PMID: 26795593

[ref107] Ho J. J. D. Balukoff N. C. Theodoridis P. R. Wang M. Krieger J. R. Schatz J. H.. (2020). A network of RNA-binding proteins controls translation efficiency to activate anaerobic metabolism. Nat. Commun. 11:2677. doi: 10.1038/s41467-020-16504-1, PMID: 32472050 PMC7260222

[ref108] Huang J. Chen X. H. Wu K. Xu P. (2005). Cloning and expression of a novel isoform of heterogeneous nuclear ribonucleoprotein-R. Neuroreport 16, 727–730. doi: 10.1097/00001756-200505120-00014, PMID: 15858414

[ref109] Huelga S. C. Vu A. Q. Arnold J. D. Liang T. Y. Liu P. P. Yan B. Y.. (2012). Integrative genome-wide analysis reveals cooperative regulation of alternative splicing by hnRNP proteins. Cell Rep. 1, 167–178. doi: 10.1016/j.celrep.2012.02.001, PMID: 22574288 PMC3345519

[ref110] Hüttelmaier S. Illenberger S. Grosheva I. Rüdiger M. Singer R. H. Jockusch B. M. (2001). Raver1, a dual compartment protein, is a ligand for PTB/hnRNPI and microfilament attachment proteins. J. Cell Biol. 155, 775–786. doi: 10.1083/jcb.200105044, PMID: 11724819 PMC2150882

[ref111] Ilik İ. Malszycki M. Lübke A. K. Schade C. Meierhofer D. Aktaş T. (2020). SON and SRRM2 are essential for nuclear speckle formation. eLife 9:e60579. doi: 10.7554/eLife.60579, PMID: 33095160 PMC7671692

[ref112] Ishigaki S. Sobue G. (2018). Importance of functional loss of FUS in FTLD/ALS. Front. Mol. Biosci. 5:44. doi: 10.3389/fmolb.2018.00044, PMID: 29774215 PMC5943504

[ref113] Izaurralde E. Jarmolowski A. Beisel C. Mattaj I. W. Dreyfuss G. Fischer U. (1997). A role for the M9 transport signal of hnRNP A1 in mRNA nuclear export. J. Cell Biol. 137, 27–35. doi: 10.1083/jcb.137.1.27, PMID: 9105034 PMC2139861

[ref114] Jagdeo J. M. Dufour A. Fung G. Luo H. Kleifeld O. Overall C. M.. (2015). Heterogeneous nuclear ribonucleoprotein M facilitates enterovirus infection. J. Virol. 89, 7064–7078. doi: 10.1128/JVI.02977-14, PMID: 25926642 PMC4473559

[ref115] Jepsen W. M. Ramsey K. Szelinger S. Llaci L. Balak C. Belnap N.. (2019). Two additional males with X-linked, syndromic mental retardation carry de novo mutations in HNRNPH2. Clin. Genet. 96, 183–185. doi: 10.1111/cge.13580, PMID: 31236915 PMC6852257

[ref116] Johansson J. Lidéus S. Frykholm C. Gunnarsson C. Mihalic F. Gudmundsson S.. (2023). Gustavson syndrome is caused by an in-frame deletion in RBMX associated with potentially disturbed SH3 domain interactions. Eur. J. Hum. Genet. 32, 253–256. doi: 10.1038/s41431-023-01498-3 37277488 PMC10923852

[ref117] Josephs K. A. Hodges J. R. Snowden J. S. Mackenzie I. R. Neumann M. Mann D. M.. (2011). Neuropathological background of phenotypical variability in frontotemporal dementia. Acta Neuropathol. 122, 137–153. doi: 10.1007/s00401-011-0839-6 21614463 PMC3232515

[ref118] Kamelgarn M. Chen J. Kuang L. Arenas A. Zhai J. Zhu H.. (2016). Proteomic analysis of FUS interacting proteins provides insights into FUS function and its role in ALS. Biochim. Biophys. Acta 1862, 2004–2014. doi: 10.1016/j.bbadis.2016.07.015, PMID: 27460707 PMC5055831

[ref119] Kamma H. Horiguchi H. Wan L. Matsui M. Fujiwara M. Fujimoto M.. (1999). Molecular characterization of the hnRNP A2/B1 proteins: tissue-specific expression and novel isoforms. Exp. Cell Res. 246, 399–411. doi: 10.1006/excr.1998.4323, PMID: 9925756

[ref120] Kamma H. Portman D. S. Dreyfuss G. (1995). Cell type-specific expression of hnRNP proteins. Exp. Cell Res. 221, 187–196. doi: 10.1006/excr.1995.1366 7589244

[ref121] Kaplanis J. Samocha K. E. Wiel L. Zhang Z. Arvai K. J. Eberhardt R. Y.. (2020). Evidence for 28 genetic disorders discovered by combining healthcare and research data. Nature 586, 757–762. doi: 10.1038/s41586-020-2832-5, PMID: 33057194 PMC7116826

[ref122] Kaur R. Batra J. Stuchlik O. Reed M. S. Pohl J. Sambhara S.. (2022). Heterogeneous ribonucleoprotein A1 (hnRNPA1) interacts with the nucleoprotein of the influenza a virus and impedes virus replication. Viruses 14:199. doi: 10.3390/v14020199, PMID: 35215793 PMC8880450

[ref123] Keating S. S. San Gil R. Swanson M. E. V. Scotter E. L. Walker A. K. (2022). TDP-43 pathology: from noxious assembly to therapeutic removal. Prog. Neurobiol. 211:102229. doi: 10.1016/j.pneurobio.2022.102229, PMID: 35101542

[ref124] Kelvington B. A. Abel T. (2023). hnRNPH2 gain-of-function mutations reveal therapeutic strategies and a role for RNA granules in neurodevelopmental disorders. J. Clin. Invest. 133:e171499. doi: 10.1172/JCI171499, PMID: 37463443 PMC10348753

[ref125] Kemmerer K. Fischer S. Weigand J. E. (2018). Auto- and cross-regulation of the hnRNPs D and DL. RNA 24, 324–331. doi: 10.1261/rna.063420.117, PMID: 29263134 PMC5824352

[ref126] Khalil B. Linsenmeier M. Smith C. L. Shorter J. Rossoll W. (2024). Nuclear-import receptors as gatekeepers of pathological phase transitions in ALS/FTD. Mol. Neurodegener. 19:8. doi: 10.1186/s13024-023-00698-1, PMID: 38254150 PMC10804745

[ref127] Khan M. I. Zhang J. Liu Q. (2021). HnRNP F and hnRNP H1 regulate mRNA stability of amyloid precursor protein. Neuroreport 32, 824–832. doi: 10.1097/WNR.0000000000001662, PMID: 33994531

[ref128] Kiledjian M. Dreyfuss G. (1992). Primary structure and binding activity of the hnRNP U protein: binding RNA through RGG box. EMBO J. 11, 2655–2664. doi: 10.1002/j.1460-2075.1992.tb05331.x, PMID: 1628625 PMC556741

[ref129] Kim T. D. Kim J. S. Kim J. H. Myung J. Chae H. D. Woo K. C.. (2005). Rhythmic serotonin N-acetyltransferase mRNA degradation is essential for the maintenance of its circadian oscillation. Mol. Cell. Biol. 25, 3232–3246. doi: 10.1128/MCB.25.8.3232-3246.2005 15798208 PMC1069600

[ref130] Kim H. J. Kim N. C. Wang Y. D. Scarborough E. A. Moore J. Diaz Z.. (2013). Mutations in prion-like domains in hnRNPA2B1 and hnRNPA1 cause multisystem proteinopathy and ALS. Nature 495, 467–473. doi: 10.1038/nature11922, PMID: 23455423 PMC3756911

[ref131] Kim D. Y. Kwak E. Kim S. H. Lee K. H. Woo K. C. Kim K. T. (2011). hnRNP Q mediates a phase-dependent translation-coupled mRNA decay of mouse Period3. Nucleic Acids Res. 39, 8901–8914. doi: 10.1093/nar/gkr605, PMID: 21785138 PMC3203584

[ref132] Kim S. H. Lee K. H. Kim D. Y. Kwak E. Kim S. Kim K. T. (2015). Rhythmic control of mRNA stability modulates circadian amplitude of mouse Period3 mRNA. J. Neurochem. 132, 642–656. doi: 10.1111/jnc.13027 25581122

[ref133] Kishor A. Ge Z. Hogg J. R. (2019). hnRNP L-dependent protection of normal mRNAs from NMD subverts quality control in B cell lymphoma. EMBO J. 38:e99128. doi: 10.15252/embj.201899128, PMID: 30530525 PMC6356069

[ref134] Klaric J. A. Wüst S. Panier S. (2021). New faces of old friends: emerging new roles of RNA-binding proteins in the DNA double-Strand break response. Front. Mol. Biosci. 8:668821. doi: 10.3389/fmolb.2021.668821, PMID: 34026839 PMC8138124

[ref135] Kleinhenz B. Fabienke M. Swiniarski S. Wittenmayer N. Kirsch J. Jockusch B. M.. (2005). Raver2, a new member of the hnRNP family. FEBS Lett. 579, 4254–4258. doi: 10.1016/j.febslet.2005.07.001, PMID: 16051233

[ref136] Klim J. R. Williams L. A. Limone F. Guerra San Juan I. Davis-Dusenbery B. N. Mordes D. A.. (2019). ALS-implicated protein TDP-43 sustains levels of STMN2, a mediator of motor neuron growth and repair. Nat. Neurosci. 22, 167–179. doi: 10.1038/s41593-018-0300-4, PMID: 30643292 PMC7153761

[ref137] Koike Y. Pickles S. Ayuso V. E. Jansen-West K. Qi Y. A. Li Z.. (2023). Correction: TDP-43 and other hnRNPs regulate cryptic exon inclusion of a key ALS/FTD risk gene, UNC13A. PLoS Biol. 21:e3002228. doi: 10.1371/journal.pbio.3002228, PMID: 37451236 PMC10348821

[ref138] Konermann S. Lotfy P. Brideau N. J. Oki J. Shokhirev M. N. Hsu P. D. (2018). Transcriptome engineering with RNA-targeting type VI-D CRISPR effectors. Cell 173, 665–676.e14. doi: 10.1016/j.cell.2018.02.033, PMID: 29551272 PMC5910255

[ref139] Korff A. Yang X. O'donovan K. Gonzalez A. Teubner B. J. Nakamura H.. (2023). A murine model of hnRNPH2-related neurodevelopmental disorder reveals a mechanism for genetic compensation by HNRNPH1. J. Clin. Invest. 133:e160309. doi: 10.1172/JCI160309 37463454 PMC10348767

[ref140] Kreienkamp H. J. Wagner M. Weigand H. Mcconkie-Rossell A. Mcdonald M. Keren B.. (2022). Variant-specific effects define the phenotypic spectrum of HNRNPH2-associated neurodevelopmental disorders in males. Hum. Genet. 141, 257–272. doi: 10.1007/s00439-021-02412-x, PMID: 34907471 PMC8807443

[ref141] Kuo P. H. Doudeva L. G. Wang Y. T. Shen C. K. Yuan H. S. (2009). Structural insights into TDP-43 in nucleic-acid binding and domain interactions. Nucleic Acids Res. 37, 1799–1808. doi: 10.1093/nar/gkp013, PMID: 19174564 PMC2665213

[ref142] Kutluay S. B. Emery A. Penumutchu S. R. Townsend D. Tenneti K. Madison M. K.. (2019). Genome-wide analysis of heterogeneous nuclear ribonucleoprotein (hnRNP) binding to HIV-1 RNA reveals a key role for hnRNP H1 in alternative viral mRNA splicing. J. Virol. 93:e01048-19. doi: 10.1128/JVI.01048-19 PMC6803249 31413137

[ref143] Kwok J. B. Loy C. T. Dobson-Stone C. Halliday G. M. (2020). The complex relationship between genotype, pathology and phenotype in familial dementia. Neurobiol. Dis. 145:105082. doi: 10.1016/j.nbd.2020.105082, PMID: 32927063

[ref144] Labranche H. Dupuis S. Ben-David Y. Bani M. R. Wellinger R. J. Chabot B. (1998). Telomere elongation by hnRNP A1 and a derivative that interacts with telomeric repeats and telomerase. Nat. Genet. 19, 199–202. doi: 10.1038/575, PMID: 9620782

[ref145] Lander E. S. Linton L. M. Birren B. Nusbaum C. Zody M. C. Baldwin J.. (2001). Initial sequencing and analysis of the human genome. Nature 409, 860–921. doi: 10.1038/35057062 11237011

[ref146] Lange L. Pagnamenta A. T. Lise S. Clasper S. Stewart H. Akha E. S.. (2016). A de novo frameshift in HNRNPK causing a kabuki-like syndrome with nodular heterotopia. Clin. Genet. 90, 258–262. doi: 10.1111/cge.12773, PMID: 26954065 PMC5006848

[ref147] Le Ber I. Van Bortel I. Nicolas G. Bouya-Ahmed K. Camuzat A. Wallon D.. (2014). hnRNPA2B1 and hnRNPA1 mutations are rare in patients with "multisystem proteinopathy" and frontotemporal lobar degeneration phenotypes. Neurobiol. Aging 35:934.e935-6. doi: 10.1016/j.neurobiolaging.2013.09.016 24119545

[ref148] Leduc M. S. Chao H. T. Qu C. Walkiewicz M. Xiao R. Magoulas P.. (2017). Clinical and molecular characterization of de novo loss of function variants in HNRNPU. Am. J. Med. Genet. A 173, 2680–2689. doi: 10.1002/ajmg.a.38388 28815871

[ref149] Lee E. K. Kim H. H. Kuwano Y. Abdelmohsen K. Srikantan S. Subaran S. S.. (2010). hnRNP C promotes APP translation by competing with FMRP for APP mRNA recruitment to P bodies. Nat. Struct. Mol. Biol. 17, 732–739. doi: 10.1038/nsmb.1815, PMID: 20473314 PMC2908492

[ref150] Lee S. Levin M. (2014). Novel somatic single nucleotide variants within the RNA binding protein hnRNP A1 in multiple sclerosis patients. F1000Res 3:132. doi: 10.12688/f1000research.4436.2, PMID: 25254102 PMC4168748

[ref151] Lee S. J. Oses-Prieto J. A. Kawaguchi R. Sahoo P. K. Kar A. N. Rozenbaum M.. (2018). hnRNPs interacting with mRNA localization motifs define axonal RNA regulons. Mol. Cell. Proteomics 17, 2091–2106. doi: 10.1074/mcp.RA118.000603, PMID: 30038033 PMC6210225

[ref152] Lelieveld S. H. Reijnders M. R. Pfundt R. Yntema H. G. Kamsteeg E. J. De Vries P.. (2016). Meta-analysis of 2,104 trios provides support for 10 new genes for intellectual disability. Nat. Neurosci. 19, 1194–1196. doi: 10.1038/nn.4352, PMID: 27479843

[ref153] Leopoldino A. M. Carregaro F. Silva C. H. Feitosa O. Mancini U. M. Freitas J. M.. (2007). Sequence and transcriptional study of HNRPK pseudogenes, and expression and molecular modeling analysis of hnRNP K isoforms. Genome 50, 451–462. doi: 10.1139/G07-016, PMID: 17612614

[ref154] Li Y. Wang H. Wan J. Ma Q. Qi Y. Gu Z. (2022). The hnRNPK/A1/R/U complex regulates gene transcription and translation and is a favorable prognostic biomarker for human colorectal adenocarcinoma. Front. Oncol. 12:845931. doi: 10.3389/fonc.2022.845931, PMID: 35875075 PMC9301189

[ref155] Li Z. Zeng W. Ye S. Lv J. Nie A. Zhang B.. (2018). Cellular hnRNP A1 interacts with nucleocapsid protein of porcine epidemic diarrhea virus and impairs viral replication. Viruses 10:127. doi: 10.3390/v10030127, PMID: 29534017 PMC5869520

[ref156] Lim M. H. Lee D. H. Jung S. E. Youn D. Y. Park C. S. Lee J. H. (2010). Effect of modulation of hnRNP L levels on the decay of bcl-2 mRNA in MCF-7 cells. Korean J. Physiol. Pharmacol. 14, 15–20. doi: 10.4196/kjpp.2010.14.1.15, PMID: 20221275 PMC2835978

[ref157] Lingenfelter P. A. Delbridge M. L. Thomas S. Hoekstra H. E. Mitchell M. J. Graves J. A.. (2001). Expression and conservation of processed copies of the RBMX gene. Mamm. Genome 12, 538–545. doi: 10.1007/s00335001-0003-z, PMID: 11420617

[ref158] Liu Y. Kim D. Choi N. Oh J. Ha J. Zhou J.. (2020). hnRNP A1 regulates alternative splicing of tau exon 10 by targeting 3' splice sites. Cells 9:936. doi: 10.3390/cells9040936, PMID: 32290247 PMC7226981

[ref159] Liu A. Q. Qin X. Wu H. Feng H. Zhang Y. A. Tu J. (2023). hnRNPA1 impedes snakehead vesiculovirus replication via competitively disrupting viral phosphoprotein-nucleoprotein interaction and degrading viral phosphoprotein. Virulence 14:2196847. doi: 10.1080/21505594.2023.2196847, PMID: 37005771 PMC10072109

[ref160] Liu G. Razanau A. Hai Y. Yu J. Sohail M. Lobo V. G.. (2012). A conserved serine of heterogeneous nuclear ribonucleoprotein L (hnRNP L) mediates depolarization-regulated alternative splicing of potassium channels. J. Biol. Chem. 287, 22709–22716. doi: 10.1074/jbc.M112.357343, PMID: 22570490 PMC3391085

[ref161] Loflin P. Chen C. Y. Shyu A. B. (1999). Unraveling a cytoplasmic role for hnRNP D in the in vivo mRNA destabilization directed by the AU-rich element. Genes Dev. 13, 1884–1897. doi: 10.1101/gad.13.14.1884, PMID: 10421639 PMC316883

[ref162] López-Erauskin J. Tadokoro T. Baughn M. W. Myers B. Mcalonis-Downes M. Chillon-Marinas C.. (2018). ALS/FTD-linked mutation in FUS suppresses intra-axonal protein synthesis and drives disease without nuclear loss-of-function of FUS. Neuron 100, 816–830.e7. doi: 10.1016/j.neuron.2018.09.044, PMID: 30344044 PMC6277851

[ref163] Low Y. H. Asi Y. Foti S. C. Lashley T. (2021). Heterogeneous nuclear ribonucleoproteins: implications in neurological diseases. Mol. Neurobiol. 58, 631–646. doi: 10.1007/s12035-020-02137-4, PMID: 33000450 PMC7843550

[ref164] Lu Y. Wang X. Gu Q. Wang J. Sui Y. Wu J.. (2022). Heterogeneous nuclear ribonucleoprotein A/B: an emerging group of cancer biomarkers and therapeutic targets. Cell Death Discov. 8:337. doi: 10.1038/s41420-022-01129-8, PMID: 35879279 PMC9314375

[ref165] Lund N. Milev M. P. Wong R. Sanmuganantham T. Woolaway K. Chabot B.. (2012). Differential effects of hnRNP D/AUF1 isoforms on HIV-1 gene expression. Nucleic Acids Res. 40, 3663–3675. doi: 10.1093/nar/gkr1238, PMID: 22187150 PMC3333888

[ref166] Ma L. Jiang Q. A. Sun L. Yang X. Huang H. Jin X.. (2020). X-linked RNA-binding motif protein modulates HIV-1 infection of CD4(+) T cells by maintaining the Trimethylation of histone H3 lysine 9 at the downstream region of the 5′ Long terminal repeat of HIV Proviral DNA. MBio 11:e03424-19. doi: 10.1128/mBio.03424-19 32317327 PMC7175097

[ref167] Ma A. S. Moran-Jones K. Shan J. Munro T. P. Snee M. J. Hoek K. S.. (2002). Heterogeneous nuclear ribonucleoprotein A3, a novel RNA trafficking response element-binding protein. J. Biol. Chem. 277, 18010–18020. doi: 10.1074/jbc.M200050200, PMID: 11886857

[ref168] Ma X. R. Prudencio M. Koike Y. Vatsavayai S. C. Kim G. Harbinski F.. (2022). TDP-43 represses cryptic exon inclusion in the FTD-ALS gene UNC13A. Nature 603, 124–130. doi: 10.1038/s41586-022-04424-7, PMID: 35197626 PMC8891019

[ref169] Makeyev A. V. Chkheidze A. N. Liebhaber S. A. (1999). A set of highly conserved RNA-binding proteins, alphaCP-1 and alphaCP-2, implicated in mRNA stabilization, are coexpressed from an intronless gene and its intron-containing paralog. J. Biol. Chem. 274, 24849–24857. doi: 10.1074/jbc.274.35.24849, PMID: 10455157

[ref170] Makeyev A. V. Kim C. B. Ruddle F. H. Enkhmandakh B. Erdenechimeg L. Bayarsaihan D. (2005). HnRNP A3 genes and pseudogenes in the vertebrate genomes. J. Exp. Zool. A Comp. Exp. Biol. 303A, 259–271. doi: 10.1002/jez.a.164 15776420

[ref171] Makeyev A. V. Liebhaber S. A. (2000). Identification of two novel mammalian genes establishes a subfamily of KH-domain RNA-binding proteins. Genomics 67, 301–316. doi: 10.1006/geno.2000.6244, PMID: 10936052

[ref172] Makeyev E. V. Zhang J. Carrasco M. A. Maniatis T. (2007). The MicroRNA miR-124 promotes neuronal differentiation by triggering brain-specific alternative pre-mRNA splicing. Mol. Cell 27, 435–448. doi: 10.1016/j.molcel.2007.07.015, PMID: 17679093 PMC3139456

[ref173] Mamontova E. M. Clément M. J. Sukhanova M. V. Joshi V. Bouhss A. Rengifo-Gonzalez J. C.. (2023). FUS RRM regulates poly(ADP-ribose) levels after transcriptional arrest and PARP-1 activation on DNA damage. Cell Rep. 42:113199. doi: 10.1016/j.celrep.2023.113199, PMID: 37804508

[ref174] Martinez-Contreras R. Cloutier P. Shkreta L. Fisette J. F. Revil T. Chabot B. (2007). hnRNP proteins and splicing control. Adv. Exp. Med. Biol. 623, 123–147. doi: 10.1007/978-0-387-77374-2_8 18380344

[ref175] Matos B. Publicover S. J. Castro L. F. C. Esteves P. J. Fardilha M. (2021). Brain and testis: more alike than previously thought? Open Biol. 11:200322. doi: 10.1098/rsob.200322, PMID: 34062096 PMC8169208

[ref176] Matsunaga S. Takata H. Morimoto A. Hayashihara K. Higashi T. Akatsuchi K.. (2012). RBMX: a regulator for maintenance and centromeric protection of sister chromatid cohesion. Cell Rep. 1, 299–308. doi: 10.1016/j.celrep.2012.02.005, PMID: 22832223

[ref177] Mayeda A. Munroe S. H. Cáceres J. F. Krainer A. R. (1994). Function of conserved domains of hnRNP A1 and other hnRNP A/B proteins. EMBO J. 13, 5483–5495. doi: 10.1002/j.1460-2075.1994.tb06883.x, PMID: 7957114 PMC395506

[ref178] Maystadt I. Deprez M. Moortgat S. Benoît V. Karadurmus D. (2020). A second case of Okamoto syndrome caused by HNRNPK mutation. Am. J. Med. Genet. A 182, 1537–1539. doi: 10.1002/ajmg.a.61568, PMID: 32222014

[ref179] Mazin P. V. Khaitovich P. Cardoso-Moreira M. Kaessmann H. (2021). Alternative splicing during mammalian organ development. Nat. Genet. 53, 925–934. doi: 10.1038/s41588-021-00851-w, PMID: 33941934 PMC8187152

[ref180] Mcglincy N. J. Tan L. Y. Paul N. Zavolan M. Lilley K. S. Smith C. W. (2010). Expression proteomics of UPF1 knockdown in HeLa cells reveals autoregulation of hnRNP A2/B1 mediated by alternative splicing resulting in nonsense-mediated mRNA decay. BMC Genomics 11:565. doi: 10.1186/1471-2164-11-565, PMID: 20946641 PMC3091714

[ref181] Mckay S. J. Cooke H. (1992). hnRNP A2/B1 binds specifically to single stranded vertebrate telomeric repeat TTAGGGn. Nucleic Acids Res. 20, 6461–6464. doi: 10.1093/nar/20.24.6461, PMID: 1282701 PMC334558

[ref182] Melamed Z. López-Erauskin J. Baughn M. W. Zhang O. Drenner K. Sun Y.. (2019). Premature polyadenylation-mediated loss of stathmin-2 is a hallmark of TDP-43-dependent neurodegeneration. Nat. Neurosci. 22, 180–190. doi: 10.1038/s41593-018-0293-z, PMID: 30643298 PMC6348009

[ref183] Miyake N. Inaba M. Mizuno S. Shiina M. Imagawa E. Miyatake S.. (2017). A case of atypical kabuki syndrome arising from a novel missense variant in HNRNPK. Clin. Genet. 92, 554–555. doi: 10.1111/cge.13023, PMID: 28771707

[ref184] Mo L. Meng L. Huang Z. Yi L. Yang N. Li G. (2022). An analysis of the role of HnRNP C dysregulation in cancers. Biomark. Res. 10:19. doi: 10.1186/s40364-022-00366-4, PMID: 35395937 PMC8994388

[ref185] Mohagheghi F. Prudencio M. Stuani C. Cook C. Jansen-West K. Dickson D. W.. (2016). TDP-43 functions within a network of hnRNP proteins to inhibit the production of a truncated human SORT1 receptor. Hum. Mol. Genet. 25, 534–545. doi: 10.1093/hmg/ddv491, PMID: 26614389 PMC4731020

[ref186] Mohanty B. K. Karam J. A. Howley B. V. Dalton A. C. Grelet S. Dincman T.. (2021). Heterogeneous nuclear ribonucleoprotein E1 binds polycytosine DNA and monitors genome integrity. Life Sci. Alliance 4:e202000995. doi: 10.26508/lsa.202000995, PMID: 34272328 PMC8321654

[ref187] Molliex A. Temirov J. Lee J. Coughlin M. Kanagaraj A. P. Kim H. J.. (2015). Phase separation by low complexity domains promotes stress granule assembly and drives pathological fibrillization. Cell 163, 123–133. doi: 10.1016/j.cell.2015.09.015, PMID: 26406374 PMC5149108

[ref188] Monette A. Ajamian L. López-Lastra M. Mouland A. J. (2009). Human immunodeficiency virus type 1 (HIV-1) induces the cytoplasmic retention of heterogeneous nuclear ribonucleoprotein A1 by disrupting nuclear import: implications for HIV-1 gene expression. J. Biol. Chem. 284, 31350–31362. doi: 10.1074/jbc.M109.048736, PMID: 19737937 PMC2781532

[ref189] Morales J. Pujar S. Loveland J. E. Astashyn A. Bennett R. Berry A.. (2022). A joint NCBI and EMBL-EBI transcript set for clinical genomics and research. Nature 604, 310–315. doi: 10.1038/s41586-022-04558-8, PMID: 35388217 PMC9007741

[ref190] Moran-Jones K. Wayman L. Kennedy D. D. Reddel R. R. Sara S. Snee M. J.. (2005). hnRNP A2, a potential ssDNA/RNA molecular adapter at the telomere. Nucleic Acids Res. 33, 486–496. doi: 10.1093/nar/gki203, PMID: 15659580 PMC548348

[ref191] Mourelatos Z. Abel L. Yong J. Kataoka N. Dreyfuss G. (2001). SMN interacts with a novel family of hnRNP and spliceosomal proteins. EMBO J. 20, 5443–5452. doi: 10.1093/emboj/20.19.5443, PMID: 11574476 PMC125643

[ref192] Moursy A. Allain F. H. Cléry A. (2014). Characterization of the RNA recognition mode of hnRNP G extends its role in SMN2 splicing regulation. Nucleic Acids Res. 42, 6659–6672. doi: 10.1093/nar/gku244, PMID: 24692659 PMC4041419

[ref193] Müller-Mcnicoll M. Rossbach O. Hui J. Medenbach J. (2019). Auto-regulatory feedback by RNA-binding proteins. J. Mol. Cell Biol. 11, 930–939. doi: 10.1093/jmcb/mjz043, PMID: 31152582 PMC6884704

[ref194] Naganuma T. Nakagawa S. Tanigawa A. Sasaki Y. F. Goshima N. Hirose T. (2012). Alternative 3′-end processing of long noncoding RNA initiates construction of nuclear paraspeckles. EMBO J. 31, 4020–4034. doi: 10.1038/emboj.2012.251, PMID: 22960638 PMC3474925

[ref195] Nedelsky N. B. Taylor J. P. (2022). Pathological phase transitions in ALS-FTD impair dynamic RNA-protein granules. RNA 28, 97–113. doi: 10.1261/rna.079001.121, PMID: 34706979 PMC8675280

[ref196] Ni J. Z. Grate L. Donohue J. P. Preston C. Nobida N. O’Brien G.. (2007). Ultraconserved elements are associated with homeostatic control of splicing regulators by alternative splicing and nonsense-mediated decay. Genes Dev. 21, 708–718. doi: 10.1101/gad.1525507, PMID: 17369403 PMC1820944

[ref197] Niggl E. Bouman A. Briere L. C. Hoogenboezem R. M. Wallaard I. Park J.. (2023). HNRNPC haploinsufficiency affects alternative splicing of intellectual disability-associated genes and causes a neurodevelopmental disorder. Am. J. Hum. Genet. 110, 1414–1435. doi: 10.1016/j.ajhg.2023.07.005, PMID: 37541189 PMC10432175

[ref198] Nishiyama H. Itoh K. Kaneko Y. Kishishita M. Yoshida O. Fujita J. (1997). A glycine-rich RNA-binding protein mediating cold-inducible suppression of mammalian cell growth. J. Cell Biol. 137, 899–908. doi: 10.1083/jcb.137.4.899, PMID: 9151692 PMC2139845

[ref199] Nolan M. Talbot K. Ansorge O. (2016). Pathogenesis of FUS-associated ALS and FTD: insights from rodent models. Acta Neuropathol. Commun. 4:99. doi: 10.1186/s40478-016-0358-8, PMID: 27600654 PMC5011941

[ref200] Nurk S. Koren S. Rhie A. Rautiainen M. Bzikadze A. V. Mikheenko A.. (2022). The complete sequence of a human genome. Science 376, 44–53. doi: 10.1126/science.abj6987, PMID: 35357919 PMC9186530

[ref201] Okamoto N. (2019). Okamoto syndrome has features overlapping with Au-Kline syndrome and is caused by HNRNPK mutation. Am. J. Med. Genet. A 179, 822–826. doi: 10.1002/ajmg.a.61079, PMID: 30793470

[ref202] Pan Q. Shai O. Lee L. J. Frey B. J. Blencowe B. J. (2008). Deep surveying of alternative splicing complexity in the human transcriptome by high-throughput sequencing. Nat. Genet. 40, 1413–1415. doi: 10.1038/ng.259, PMID: 18978789

[ref203] Papadopoulou C. Boukakis G. Ganou V. Patrinou-Georgoula M. Guialis A. (2012). Expression profile and interactions of hnRNP A3 within hnRNP/mRNP complexes in mammals. Arch. Biochem. Biophys. 523, 151–160. doi: 10.1016/j.abb.2012.04.012, PMID: 22546510

[ref204] Peron A. Novara F. La Briola F. Merati E. Giannusa E. Segalini E.. (2020). Missense variants in the Arg206 residue of HNRNPH2: further evidence of causality and expansion of the phenotype. Am. J. Med. Genet. A 182, 823–828. doi: 10.1002/ajmg.a.61486, PMID: 31943778

[ref205] Pettit Kneller E. L. Connor J. H. Lyles D. S. (2009). hnRNPs Relocalize to the cytoplasm following infection with vesicular stomatitis virus. J. Virol. 83, 770–780. doi: 10.1128/JVI.01279-08, PMID: 19004954 PMC2612367

[ref206] Pickering B. M. Mitchell S. A. Evans J. R. Willis A. E. (2003). Polypyrimidine tract binding protein and poly r(C) binding protein 1 interact with the BAG-1 IRES and stimulate its activity in vitro and in vivo. Nucleic Acids Res. 31, 639–646. doi: 10.1093/nar/gkg146, PMID: 12527772 PMC140511

[ref207] Pilch J. Koppolu A. A. Walczak A. Murcia Pienkowski V. A. Biernacka A. Skiba P.. (2018). Evidence for HNRNPH1 being another gene for Bain type syndromic mental retardation. Clin. Genet. 94, 381–385. doi: 10.1111/cge.13410 29938792

[ref208] Piñol-Roma S. (1997). HnRNP proteins and the nuclear export of mRNA. Semin. Cell Dev. Biol. 8, 57–63. doi: 10.1006/scdb.1996.0122 15001106

[ref209] Piñol-Roma S. Choi Y. D. Matunis M. J. Dreyfuss G. (1988). Immunopurification of heterogeneous nuclear ribonucleoprotein particles reveals an assortment of RNA-binding proteins. Genes Dev. 2, 215–227. doi: 10.1101/gad.2.2.215 3129338

[ref210] Piñol-Roma S. Dreyfuss G. (1992). Shuttling of pre-mRNA binding proteins between nucleus and cytoplasm. Nature 355, 730–732. doi: 10.1038/355730a0 1371331

[ref211] Provasek V. E. Mitra J. Malojirao V. H. Hegde M. L. (2022). DNA double-Strand breaks as pathogenic lesions in neurological disorders. Int. J. Mol. Sci. 23:4653. doi: 10.3390/ijms23094653, PMID: 35563044 PMC9099445

[ref212] Purice M. D. Taylor J. P. (2018). Linking hnRNP function to ALS and FTD pathology. Front. Neurosci. 12:326. doi: 10.3389/fnins.2018.00326, PMID: 29867335 PMC5962818

[ref213] Rahman M. A. Masuda A. Ohe K. Ito M. Hutchinson D. O. Mayeda A.. (2013). HnRNP L and hnRNP LL antagonistically modulate PTB-mediated splicing suppression of CHRNA1 pre-mRNA. Sci. Rep. 3:2931. doi: 10.1038/srep02931, PMID: 24121633 PMC3796306

[ref214] Rajagopalan L. E. Westmark C. J. Jarzembowski J. A. Malter J. S. (1998). hnRNP C increases amyloid precursor protein (APP) production by stabilizing APP mRNA. Nucleic Acids Res. 26, 3418–3423. doi: 10.1093/nar/26.14.3418, PMID: 9649628 PMC147701

[ref215] Rauch A. Wieczorek D. Graf E. Wieland T. Endele S. Schwarzmayr T.. (2012). Range of genetic mutations associated with severe non-syndromic sporadic intellectual disability: an exome sequencing study. Lancet 380, 1674–1682. doi: 10.1016/S0140-6736(12)61480-9, PMID: 23020937

[ref216] Reber S. Stettler J. Filosa G. Colombo M. Jutzi D. Lenzken S. C.. (2016). Minor intron splicing is regulated by FUS and affected by ALS-associated FUS mutants. EMBO J. 35, 1504–1521. doi: 10.15252/embj.201593791, PMID: 27252488 PMC4946139

[ref217] Redondo N. Madan V. Alvarez E. Carrasco L. (2015). Impact of vesicular stomatitis virus M proteins on different cellular functions. PLoS One 10:e0131137. doi: 10.1371/journal.pone.0131137, PMID: 26091335 PMC4474437

[ref218] Reichert S. C. Li R. Turner S. A. van Jaarsveld R. H. Massink M. P. G. van den Boogaard M. J. H.. (2020). HNRNPH1-related syndromic intellectual disability: seven additional cases suggestive of a distinct syndromic neurodevelopmental syndrome. Clin. Genet. 98, 91–98. doi: 10.1111/cge.13765, PMID: 32335897

[ref219] Resnick M. Segall A. G G. R. K. Lupowitz Z. Zisapel N. (2008). Alternative splicing of neurexins: a role for neuronal polypyrimidine tract binding protein. Neurosci. Lett. 439, 235–240. doi: 10.1016/j.neulet.2008.05.034, PMID: 18534753

[ref220] Reznik B. Clement S. L. Lykke-Andersen J. (2014). hnRNP F complexes with tristetraprolin and stimulates ARE-mRNA decay. PLoS One 9:e100992. doi: 10.1371/journal.pone.0100992, PMID: 24978456 PMC4076271

[ref221] Romanelli M. G. Lorenzi P. Morandi C. (2000). Organization of the human gene encoding heterogeneous nuclear ribonucleoprotein type I (hnRNP I) and characterization of hnRNP I related pseudogene. Gene 255, 267–272. doi: 10.1016/S0378-1119(00)00331-0, PMID: 11024286

[ref222] Romano M. Buratti E. (2013). Targeting RNA binding proteins involved in neurodegeneration. J. Biomol. Screen. 18, 967–983. doi: 10.1177/1087057113497256 23954928

[ref223] Romero F. Germani A. Puvion E. Camonis J. Varin-Blank N. Gisselbrecht S.. (1998). Vav binding to heterogeneous nuclear ribonucleoprotein (hnRNP) C. Evidence for Vav-hnRNP interactions in an RNA-dependent manner. J. Biol. Chem. 273, 5923–5931. doi: 10.1074/jbc.273.10.5923 9488731

[ref224] Rossbach O. Hung L. H. Schreiner S. Grishina I. Heiner M. Hui J.. (2009). Auto- and cross-regulation of the hnRNP L proteins by alternative splicing. Mol. Cell. Biol. 29, 1442–1451. doi: 10.1128/MCB.01689-08, PMID: 19124611 PMC2648227

[ref225] Sakakibara S. Nakamura Y. Satoh H. Okano H. (2001). RNA-binding protein Musashi2: developmentally regulated expression in neural precursor cells and subpopulations of neurons in mammalian CNS. J. Neurosci. 21, 8091–8107. doi: 10.1523/JNEUROSCI.21-20-08091.2001, PMID: 11588182 PMC6763847

[ref226] Salapa H. E. Hutchinson C. Popescu B. F. Levin M. C. (2020). Neuronal RNA-binding protein dysfunction in multiple sclerosis cortex. Ann. Clin. Transl. Neurol. 7, 1214–1224. doi: 10.1002/acn3.51103, PMID: 32608162 PMC7359129

[ref227] Salapa H. E. Thibault P. A. Libner C. D. Ding Y. Clarke J. W. E. Denomy C.. (2024). hnRNP A1 dysfunction alters RNA splicing and drives neurodegeneration in multiple sclerosis (MS). Nat. Commun. 15:356. doi: 10.1038/s41467-023-44658-1, PMID: 38191621 PMC10774274

[ref228] Salehi S. Zare A. Prezza G. Bader J. Schneider C. Fischer U.. (2023). Cytosolic Ptbp2 modulates axon growth in motoneurons through axonal localization and translation of *Hnrnpr*. Nat. Commun. 14:4158. doi: 10.1038/s41467-023-39787-6 37438340 PMC10338680

[ref229] Sama R. R. Fallini C. Gatto R. Mckeon J. E. Song Y. Rotunno M. S.. (2017). ALS-linked FUS exerts a gain of toxic function involving aberrant p38 MAPK activation. Sci. Rep. 7:115. doi: 10.1038/s41598-017-00091-1, PMID: 28273913 PMC5428330

[ref230] Sapir T. Kshirsagar A. Gorelik A. Olender T. Porat Z. Scheffer I. E.. (2022). Heterogeneous nuclear ribonucleoprotein U (HNRNPU) safeguards the developing mouse cortex. Nat. Commun. 13:4209. doi: 10.1038/s41467-022-31752-z, PMID: 35864088 PMC9304408

[ref231] Scalabrin M. Frasson I. Ruggiero E. Perrone R. Tosoni E. Lago S.. (2017). The cellular protein hnRNP A2/B1 enhances HIV-1 transcription by unfolding LTR promoter G-quadruplexes. Sci. Rep. 7:45244. doi: 10.1038/srep45244, PMID: 28338097 PMC5364415

[ref232] Scekic-Zahirovic J. Sendscheid O. El Oussini H. Jambeau M. Sun Y. Mersmann S.. (2016). Toxic gain of function from mutant FUS protein is crucial to trigger cell autonomous motor neuron loss. EMBO J. 35, 1077–1097. doi: 10.15252/embj.201592559, PMID: 26951610 PMC4868956

[ref233] Seczynska M. Lehner P. J. (2023). The sound of silence: mechanisms and implications of HUSH complex function. Trends Genet. 39, 251–267. doi: 10.1016/j.tig.2022.12.005, PMID: 36754727

[ref234] Semino F. Schröter J. Willemsen M. H. Bast T. Biskup S. Beck-Woedl S.. (2021). Further evidence for de novo variants in SYNCRIP as the cause of a neurodevelopmental disorder. Hum. Mutat. 42, 1094–1100. doi: 10.1002/humu.24245 34157790

[ref235] Sharma A. Lyashchenko A. K. Lu L. Nasrabady S. E. Elmaleh M. Mendelsohn M.. (2016). ALS-associated mutant FUS induces selective motor neuron degeneration through toxic gain of function. Nat. Commun. 7:10465. doi: 10.1038/ncomms10465, PMID: 26842965 PMC4742863

[ref236] Shashi V. Berry M. N. Shoaf S. Sciote J. J. Goldstein D. Hart T. C. (2000). A unique form of mental retardation with a distinctive phenotype maps to Xq26-q27. Am. J. Hum. Genet. 66, 469–479. doi: 10.1086/302772, PMID: 10677307 PMC1288100

[ref237] Shashi V. Xie P. Schoch K. Goldstein D. B. Howard T. D. Berry M. N.. (2015). The RBMX gene as a candidate for the Shashi X-linked intellectual disability syndrome. Clin. Genet. 88, 386–390. doi: 10.1111/cge.12511, PMID: 25256757

[ref238] Sheikh M. S. Carrier F. Papathanasiou M. A. Hollander M. C. Zhan Q. Yu K.. (1997). Identification of several human homologs of hamster DNA damage-inducible transcripts. Cloning and characterization of a novel UV-inducible cDNA that codes for a putative RNA-binding protein. J. Biol. Chem. 272, 26720–26726. doi: 10.1074/jbc.272.42.26720, PMID: 9334257

[ref239] Siachisumo C. Luzzi S. Aldalaqan S. Hysenaj G. Dalgliesh C. Cheung K.. (2023). An anciently diverged family of RNA binding proteins maintain correct splicing of a class of ultra-long exons through cryptic splice site repression: Cold Spring Harbor Laboratory. 10.7554/eLife.89705 PMC11446547 39356106

[ref240] Siddiqui A. Saxena A. Echols J. Havasi V. Fu L. Keeling K. M. (2023). RNA binding proteins PTBP1 and HNRNPL regulate CFTR mRNA decay. Heliyon 9:e22281. doi: 10.1016/j.heliyon.2023.e22281, PMID: 38045134 PMC10692906

[ref241] Sidhu R. Gatt A. Fratta P. Lashley T. Bampton A. (2022). HnRNP K mislocalisation in neurons of the dentate nucleus is a novel neuropathological feature of neurodegenerative disease and ageing. Neuropathol. Appl. Neurobiol. 48:e12793. doi: 10.1111/nan.12793, PMID: 35064577 PMC9208575

[ref242] Simard M. J. Chabot B. (2002). SRp30c is a repressor of 3′ splice site utilization. Mol. Cell. Biol. 22, 4001–4010. doi: 10.1128/MCB.22.12.4001-4010.2002, PMID: 12024014 PMC133842

[ref243] Singh R. N. Singh N. N. (2018). Mechanism of splicing regulation of spinal muscular atrophy genes. Adv. Neurobiol. 20, 31–61. doi: 10.1007/978-3-319-89689-2_2 29916015 PMC6026014

[ref244] Singh R. Valcárcel J. (2005). Building specificity with nonspecific RNA-binding proteins. Nat. Struct. Mol. Biol. 12, 645–653. doi: 10.1038/nsmb961, PMID: 16077728

[ref245] Siomi H. Dreyfuss G. (1995). A nuclear localization domain in the hnRNP A1 protein. J. Cell Biol. 129, 551–560. doi: 10.1083/jcb.129.3.551, PMID: 7730395 PMC2120450

[ref246] Smith S. A. Ray D. Cook K. B. Mallory M. J. Hughes T. R. Lynch K. W. (2013). Paralogs hnRNP L and hnRNP LL exhibit overlapping but distinct RNA binding constraints. PLoS One 8:e80701. doi: 10.1371/journal.pone.0080701, PMID: 24244709 PMC3823766

[ref247] Somashekar P. H. Narayanan D. L. Jagadeesh S. Suresh B. Vaishnavi R. D. Bielas S.. (2020). Bain type of X-linked syndromic mental retardation in a male with a pathogenic variant in HNRNPH2. Am. J. Med. Genet. A 182, 183–188. doi: 10.1002/ajmg.a.61388, PMID: 31670473 PMC10052778

[ref248] Soniat M. Chook Y. M. (2016). Karyopherin-β2 recognition of a PY-NLS variant that lacks the proline-tyrosine motif. Structure 24, 1802–1809. doi: 10.1016/j.str.2016.07.018, PMID: 27618664 PMC5053885

[ref249] Spellman R. Llorian M. Smith C. W. (2007). Crossregulation and functional redundancy between the splicing regulator PTB and its paralogs nPTB and ROD1. Mol. Cell 27, 420–434. doi: 10.1016/j.molcel.2007.06.016, PMID: 17679092 PMC1940037

[ref250] Sreedharan J. Blair I. P. Tripathi V. B. Hu X. Vance C. Rogelj B.. (2008). TDP-43 mutations in familial and sporadic amyotrophic lateral sclerosis. Science 319, 1668–1672. doi: 10.1126/science.1154584, PMID: 18309045 PMC7116650

[ref251] Stake M. Singh D. Singh G. Marcela Hernandez J. Kaddis Maldonado R. Parent L. J.. (2015). HIV-1 and two avian retroviral 5′ untranslated regions bind orthologous human and chicken RNA binding proteins. Virology 486, 307–320. doi: 10.1016/j.virol.2015.06.001, PMID: 26584240 PMC4877169

[ref252] Stelzer G. Rosen N. Plaschkes I. Zimmerman S. Twik M. Fishilevich S.. (2016). The GeneCards suite: from gene data mining to disease genome sequence analyses. Curr. Protoc. Bioinformatics 54, 1.30.31–31.30.33. doi: 10.1002/cpbi.5 27322403

[ref253] Sueoka E. Yukitake M. Iwanaga K. Sueoka N. Aihara T. Kuroda Y. (2004). Autoantibodies against heterogeneous nuclear ribonucleoprotein B1 in CSF of MS patients. Ann. Neurol. 56, 778–786. doi: 10.1002/ana.20276, PMID: 15497154

[ref254] Sun S. Ling S. C. Qiu J. Albuquerque C. P. Zhou Y. Tokunaga S.. (2015). ALS-causative mutations in FUS/TLS confer gain and loss of function by altered association with SMN and U1-snRNP. Nat. Commun. 6:6171. doi: 10.1038/ncomms7171, PMID: 25625564 PMC4338613

[ref255] Suzuki H. Matsuoka M. (2015). Overexpression of nuclear FUS induces neuronal cell death. Neuroscience 287, 113–124. doi: 10.1016/j.neuroscience.2014.12.007, PMID: 25497700

[ref256] Suzuki N. Nishiyama A. Warita H. Aoki M. (2023). Genetics of amyotrophic lateral sclerosis: seeking therapeutic targets in the era of gene therapy. J. Hum. Genet. 68, 131–152. doi: 10.1038/s10038-022-01055-8, PMID: 35691950 PMC9968660

[ref257] Tanaka E. Fukuda H. Nakashima K. Tsuchiya N. Seimiya H. Nakagama H. (2007). HnRNP A3 binds to and protects mammalian telomeric repeats in vitro. Biochem. Biophys. Res. Commun. 358, 608–614. doi: 10.1016/j.bbrc.2007.04.177, PMID: 17502110

[ref258] Taylor J. Spiller M. Ranguin K. Vitobello A. Philippe C. Bruel A. L.. (2022). Expanding the phenotype of HNRNPU-related neurodevelopmental disorder with emphasis on seizure phenotype and review of literature. Am. J. Med. Genet. A 188, 1497–1514. doi: 10.1002/ajmg.a.62677, PMID: 35138025 PMC9305207

[ref259] Telley L. Agirman G. Prados J. Amberg N. Fièvre S. Oberst P.. (2019). Temporal patterning of apical progenitors and their daughter neurons in the developing neocortex. Science 364:eaav2522. doi: 10.1126/science.aav2522, PMID: 31073041

[ref260] Tsai Y. L. Coady T. H. Lu L. Zheng D. Alland I. Tian B.. (2020). ALS/FTD-associated protein FUS induces mitochondrial dysfunction by preferentially sequestering respiratory chain complex mRNAs. Genes Dev. 34, 785–805. doi: 10.1101/gad.335836.119, PMID: 32381627 PMC7263147

[ref261] Tuersun H. Liu L. Zhang J. Maimaitizunong R. Tang X. Li H. (2023). m6A reading protein RBMX as a biomarker for prognosis and tumor progression in esophageal cancer. Transl. Cancer Res. 12, 2319–2335. doi: 10.21037/tcr-23-84, PMID: 37859733 PMC10583014

[ref262] Van Langenhove T. Van Der Zee J. Van Broeckhoven C. (2012). The molecular basis of the frontotemporal lobar degeneration-amyotrophic lateral sclerosis spectrum. Ann. Med. 44, 817–828. doi: 10.3109/07853890.2012.665471, PMID: 22420316 PMC3529157

[ref263] Van Lindt J. Lazar T. Pakravan D. Demulder M. Meszaros A. Van Den Bosch L.. (2022). F/YGG-motif is an intrinsically disordered nucleic-acid binding motif. RNA Biol. 19, 622–635. doi: 10.1080/15476286.2022.2066336, PMID: 35491929 PMC9067507

[ref264] Venables J. P. Elliott D. J. Makarova O. V. Makarov E. M. Cooke H. J. Eperon I. C. (2000). RBMY, a probable human spermatogenesis factor, and other hnRNP G proteins interact with Tra2beta and affect splicing. Hum. Mol. Genet. 9, 685–694. doi: 10.1093/hmg/9.5.685, PMID: 10749975

[ref265] Venter J. C. Adams M. D. Myers E. W. Li P. W. Mural R. J. Sutton G. G.. (2001). The sequence of the human genome. Science 291, 1304–1351. doi: 10.1126/science.1058040 11181995

[ref266] Vollger M. R. Dishuck P. C. Sorensen M. Welch A. E. Dang V. Dougherty M. L.. (2019). Long-read sequence and assembly of segmental duplications. Nat. Methods 16, 88–94. doi: 10.1038/s41592-018-0236-3, PMID: 30559433 PMC6382464

[ref267] Vuong J. K. Lin C. H. Zhang M. Chen L. Black D. L. Zheng S. (2016). PTBP1 and PTBP2 serve Both specific and redundant functions in neuronal pre-mRNA splicing. Cell Rep. 17, 2766–2775. doi: 10.1016/j.celrep.2016.11.034, PMID: 27926877 PMC5179036

[ref268] Wall M. L. Bera A. Wong F. K. Lewis S. M. (2020). Cellular stress orchestrates the localization of hnRNP H to stress granules. Exp. Cell Res. 394:112111. doi: 10.1016/j.yexcr.2020.112111, PMID: 32473225

[ref269] Wang E. Aslanzadeh V. Papa F. Zhu H. De La Grange P. Cambi F. (2012). Global profiling of alternative splicing events and gene expression regulated by hnRNPH/F. PLoS One 7:e51266. doi: 10.1371/journal.pone.0051266, PMID: 23284676 PMC3524136

[ref270] Wang E. Dimova N. Cambi F. (2007). PLP/DM20 ratio is regulated by hnRNPH and F and a novel G-rich enhancer in oligodendrocytes. Nucleic Acids Res. 35, 4164–4178. doi: 10.1093/nar/gkm387, PMID: 17567613 PMC1919487

[ref271] Wang E. T. Sandberg R. Luo S. Khrebtukova I. Zhang L. Mayr C.. (2008). Alternative isoform regulation in human tissue transcriptomes. Nature 456, 470–476. doi: 10.1038/nature07509, PMID: 18978772 PMC2593745

[ref272] Wang J. Sun D. Wang M. Cheng A. Zhu Y. Mao S.. (2022). Multiple functions of heterogeneous nuclear ribonucleoproteins in the positive single-stranded RNA virus life cycle. Front. Immunol. 13:989298. doi: 10.3389/fimmu.2022.989298, PMID: 36119073 PMC9478383

[ref273] Weighardt F. Biamonti G. Riva S. (1995). Nucleo-cytoplasmic distribution of human hnRNP proteins: a search for the targeting domains in hnRNP A1. J. Cell Sci. 108, 545–555. doi: 10.1242/jcs.108.2.545, PMID: 7769000

[ref274] White E. J. Brewer G. Wilson G. M. (2013). Post-transcriptional control of gene expression by AUF1: mechanisms, physiological targets, and regulation. Biochim. Biophys. Acta 1829, 680–688. doi: 10.1016/j.bbagrm.2012.12.002, PMID: 23246978 PMC3664190

[ref275] Wiesmann N. Strozynski J. Beck C. Zimmermann N. Mendler S. Gieringer R.. (2017). Knockdown of hnRNPK leads to increased DNA damage after irradiation and reduces survival of tumor cells. Carcinogenesis 38, 321–328. doi: 10.1093/carcin/bgx006, PMID: 28426877

[ref276] Wilkinson M. E. Charenton C. Nagai K. (2020). RNA splicing by the spliceosome. Annu. Rev. Biochem. 89, 359–388. doi: 10.1146/annurev-biochem-091719-064225 31794245

[ref277] Wirth B. (2021). Spinal muscular atrophy: in the challenge lies a solution. Trends Neurosci. 44, 306–322. doi: 10.1016/j.tins.2020.11.009, PMID: 33423791

[ref278] Wirth B. Karakaya M. Kye M. J. Mendoza-Ferreira N. (2020). Twenty-five years of spinal muscular atrophy research: from phenotype to genotype to therapy, and what comes next. Annu. Rev. Genomics Hum. Genet. 21, 231–261. doi: 10.1146/annurev-genom-102319-103602, PMID: 32004094

[ref279] Wollerton M. C. Gooding C. Wagner E. J. Garcia-Blanco M. A. Smith C. W. (2004). Autoregulation of polypyrimidine tract binding protein by alternative splicing leading to nonsense-mediated decay. Mol. Cell 13, 91–100. doi: 10.1016/S1097-2765(03)00502-1, PMID: 14731397

[ref280] Woo K. C. Ha D. C. Lee K. H. Kim D. Y. Kim T. D. Kim K. T. (2010). Circadian amplitude of cryptochrome 1 is modulated by mRNA stability regulation via cytoplasmic hnRNP D oscillation. Mol. Cell. Biol. 30, 197–205. doi: 10.1128/MCB.01154-09, PMID: 19858287 PMC2798294

[ref281] Woo K. C. Kim T. D. Lee K. H. Kim D. Y. Kim W. Lee K. Y.. (2009). Mouse period 2 mRNA circadian oscillation is modulated by PTB-mediated rhythmic mRNA degradation. Nucleic Acids Res. 37, 26–37. doi: 10.1093/nar/gkn893, PMID: 19010962 PMC2615616

[ref282] Wood A. Gurfinkel Y. Polain N. Lamont W. Lyn Rea S. (2021). Molecular mechanisms underlying TDP-43 pathology in cellular and animal models of ALS and FTLD. Int. J. Mol. Sci. 22:4705. doi: 10.3390/ijms22094705, PMID: 33946763 PMC8125728

[ref283] Woolaway K. Asai K. Emili A. Cochrane A. (2007). hnRNP E1 and E2 have distinct roles in modulating HIV-1 gene expression. Retrovirology 4:28. doi: 10.1186/1742-4690-4-28, PMID: 17451601 PMC1863430

[ref284] Xu N. Chen C. Y. Shyu A. B. (2001). Versatile role for hnRNP D isoforms in the differential regulation of cytoplasmic mRNA turnover. Mol. Cell. Biol. 21, 6960–6971. doi: 10.1128/MCB.21.20.6960-6971.2001, PMID: 11564879 PMC99872

[ref285] Yang L. Gal J. Chen J. Zhu H. (2014). Self-assembled FUS binds active chromatin and regulates gene transcription. Proc. Natl. Acad. Sci. USA 111, 17809–17814. doi: 10.1073/pnas.1414004111, PMID: 25453086 PMC4273402

[ref286] Yates T. M. Vasudevan P. C. Chandler K. E. Donnelly D. E. Stark Z. Sadedin S.. (2017). De novo mutations in HNRNPU result in a neurodevelopmental syndrome. Am. J. Med. Genet. A 173, 3003–3012. doi: 10.1002/ajmg.a.38492, PMID: 28944577 PMC6555908

[ref287] Yeap B. B. Voon D. C. Vivian J. P. Mcculloch R. K. Thomson A. M. Giles K. M.. (2002). Novel binding of HuR and poly(C)-binding protein to a conserved UC-rich motif within the 3′-untranslated region of the androgen receptor messenger RNA. J. Biol. Chem. 277, 27183–27192. doi: 10.1074/jbc.M202883200, PMID: 12011088

[ref288] Yu J. Hai Y. Liu G. Fang T. Kung S. K. Xie J. (2009). The heterogeneous nuclear ribonucleoprotein L is an essential component in the Ca2+/calmodulin-dependent protein kinase IV-regulated alternative splicing through cytidine-adenosine repeats. J. Biol. Chem. 284, 1505–1513. doi: 10.1074/jbc.M805113200, PMID: 19017650 PMC3471988

[ref289] Yugami M. Kabe Y. Yamaguchi Y. Wada T. Handa H. (2007). hnRNP-U enhances the expression of specific genes by stabilizing mRNA. FEBS Lett. 581, 1–7. doi: 10.1016/j.febslet.2006.11.062, PMID: 17174306 PMC7130276

[ref290] Zhang X. Chen M. H. Wu X. Kodani A. Fan J. Doan R.. (2016). Cell-type-specific alternative splicing governs cell fate in the developing cerebral cortex. Cell 166, 1147–1162.e15. doi: 10.1016/j.cell.2016.07.025 27565344 PMC5248659

[ref291] Zhang M. Ergin V. Lin L. Stork C. Chen L. Zheng S. (2019). Axonogenesis is coordinated by neuron-specific alternative splicing programming and splicing regulator PTBP2. Neuron 101, 690–706.e10. doi: 10.1016/j.neuron.2019.01.022, PMID: 30733148 PMC6474845

[ref292] Zhang Q. Ma C. Gearing M. Wang P. G. Chin L. S. Li L. (2018). Integrated proteomics and network analysis identifies protein hubs and network alterations in Alzheimer's disease. Acta Neuropathol. Commun. 6:19. doi: 10.1186/s40478-018-0524-2, PMID: 29490708 PMC5831854

[ref293] Zhang Q. Zhang J. Ye J. Li X. Liu H. Ma X.. (2021). Nuclear speckle specific hnRNP D-like prevents age- and AD-related cognitive decline by modulating RNA splicing. Mol. Neurodegener. 16:66. doi: 10.1186/s13024-021-00485-w, PMID: 34551807 PMC8456587

[ref294] Zheng S. Gray E. E. Chawla G. Porse B. T. O'Dell T. J. Black D. L. (2012). PSD-95 is post-transcriptionally repressed during early neural development by PTBP1 and PTBP2. Nat. Neurosci. 15:s381, 381–388. doi: 10.1038/nn.3026, PMID: 22246437 PMC3288398

[ref295] Zheng T. Zhou H. Li X. Peng D. Yang Y. Zeng Y.. (2020). RBMX is required for activation of ATR on repetitive DNAs to maintain genome stability. Cell Death Differ. 27, 3162–3176. doi: 10.1038/s41418-020-0570-8, PMID: 32494026 PMC7560680

[ref296] Zhou J. Guo Y. Huo Z. Xing Y. Fang J. Ma G.. (2021). Identification of therapeutic targets and prognostic biomarkers from the hnRNP family in invasive breast carcinoma. Aging (Albany NY) 13, 4503–4521. doi: 10.18632/aging.202411, PMID: 33495416 PMC7906176

